# Gene expression signatures of human cell and tissue longevity

**DOI:** 10.1038/npjamd.2016.14

**Published:** 2016-07-07

**Authors:** Inge Seim, Siming Ma, Vadim N Gladyshev

**Affiliations:** 1 Division of Genetics, Department of Medicine, Brigham and Women’s Hospital and Harvard Medical School, Boston, MA, USA

## Abstract

Different cell types within the body exhibit substantial variation in the average time they live, ranging from days to the lifetime of the organism. The underlying mechanisms governing the diverse lifespan of different cell types are not well understood. To examine gene expression strategies that support the lifespan of different cell types within the human body, we obtained publicly available RNA-seq data sets and interrogated transcriptomes of 21 somatic cell types and tissues with reported cellular turnover, a *bona fide* estimate of lifespan, ranging from 2 days (monocytes) to a lifetime (neurons). Exceptionally long-lived neurons presented a gene expression profile of reduced protein metabolism, consistent with neuronal survival and similar to expression patterns induced by longevity interventions such as dietary restriction. Across different cell lineages, we identified a gene expression signature of human cell and tissue turnover. In particular, turnover showed a negative correlation with the energetically costly cell cycle and factors supporting genome stability, concomitant risk factors for aging-associated pathologies. In addition, the expression of p53 was negatively correlated with cellular turnover, suggesting that low p53 activity supports the longevity of post-mitotic cells with inherently low risk of developing cancer. Our results demonstrate the utility of comparative approaches in unveiling gene expression differences among cell lineages with diverse cell turnover within the same organism, providing insights into mechanisms that could regulate cell longevity.

## Introduction

Nature can achieve exceptional organismal longevity, >100 years in the case of humans. However, there is substantial variation in ‘cellular lifespan’, which can be conceptualized as the turnover of individual cell lineages within an individual organism.^1^ Turnover is defined as a balance between cell proliferation and death that contributes to cell and tissue homeostasis.^2^ For example, the integrity of the heart and brain is largely maintained by cells with low turnover/long lifespan, while other organs and tissues, such as the outer layers of the skin and blood cells, rely on high cell turnover/short lifespan.^3–5^ Variation in cellular lifespan is also evident across lineages derived from the same germ layers formed during embryogenesis. For example, the ectoderm gives rise to both long-lived neurons^4,6,7^ and short-lived epidermal skin cells.^8^ Similarly, the mesoderm gives rise to long-lived skeletal muscle^4^ and heart muscle^9^ and short-lived monocytes,^10,11^ while the endoderm is the origin of long-lived thyrocytes (cells of the thyroid gland)^12^ and short-lived urinary bladder cells.^13^

How such diverse cell lineage lifespans are supported within a single organism is not clear, but it appears that differentiation shapes lineages through epigenetic changes to establish biological strategies that give rise to lifespans that support the best fitness for cells in their respective niche. As fitness is subject to trade-offs, different cell types will adjust their gene regulatory networks according to their lifespan. We are interested in gene expression signatures that support diverse biological strategies to achieve longevity. Prior work on species longevity can help inform strategies for tackling this research question. Species longevity is a product of evolution and is largely shaped by genetic and environmental factors.^14^ Comparative transcriptome studies of long-lived and short-lived mammals, and analyses that examined the longevity trait across a large group of mammals (tissue-by-tissue surveys, focusing on brain, liver and kidney), have revealed candidate longevity-associated processes.^15,16^ They provide gene expression signatures of longevity across mammals and may inform on interventions that mimic these changes, thereby potentially extending lifespan. It then follows that, in principle, comparative analyses of different cell types and tissues of a single organism may similarly reveal lifespan-promoting genes and pathways. Such analyses across cell types would be conceptually similar, yet orthogonal, to the analysis across species. Publicly available transcriptome data sets (for example, RNA-seq) generated by consortia, such as the Human Protein Atlas (HPA),^17^ Encyclopedia of DNA Elements (ENCODE),^18^ Functional Annotation Of Mammalian genome (FANTOM)^19^ and the Genotype-Tissue Expression (GTEx) project,^20^ are now available. They offer an opportunity to understand how gene expression programs are related to cellular turnover, as a proxy for cellular lifespan. Here we examined transcriptomes of 21 somatic cells and tissues to assess the utility of comparative gene expression methods for the identification of longevity-associated gene signatures.

## Results

**Figure 1:**
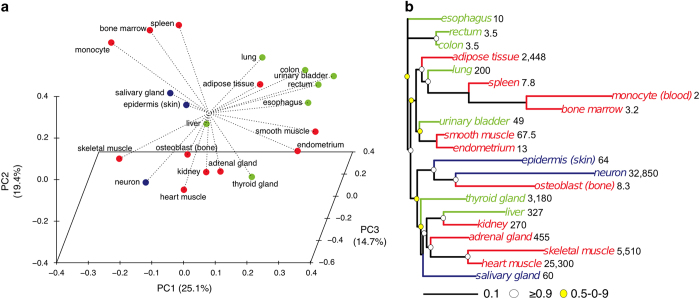
Clustering of gene expression from diverse human cells and tissues. (**a**) Representations of gene expression in Principal Component (PC) space. Values in parenthesis indicate the percentage of variance explained by each of the PCs. Ostensible germ layer origin is indicated (red: mesoderm; blue: ectoderm, green: endoderm). (**b**) Gene expression tree. Branches are colored according to germ layer origin (as in **a**). Estimated cell and tissue turnover (‘cellular lifespan’; in days) is shown next to cell and tissue names. The tree was generated by neighbor-joining (NJ) method (bootstrap=1,000,000). Bootstrap values are indicated by circles: white ⩾0.9; yellow ⩽0.9.

We interrogated publicly available transcriptomes (paired-end RNA-seq reads) of 21 human cell types and tissues, comprising 153 individual samples, with a mean age of 56 years (Table 1; details in Supplementary Table S1). Their turnover rates (an estimate of cell lifespan^4^) varied from 2 (monocytes) to 32,850 (neurons) days, with all three germ layers giving rise to both short-lived and long-lived cell lineages. Biological replicates showed Pearson's correlation coefficients above 0.90, indicating reproducibility of the gene expression data (Supplementary Table S2; Supplementary Figure S1). Gene expression patterns were first analyzed by Principal Component Analysis (PCA) (Figure 1a), which revealed that the first three Principal Components (PCs) accounted for ~60% of gene expression variation. The cells and tissues formed several clusters, suggesting related biological functions for these clusters. For example, the gastrointestinal tissues, esophagus, rectum and colon all grouped together, and hematopoietic tissues (bone marrow and spleen) and monocytes also clustered. Because transcriptomes of functionally related cell types often exhibit substantial hierarchical structure,^21,22^ a neighbor-joining gene expression tree was generated based on mean gene expression levels (Figure 1b). Similar to the PCA results, bone marrow and spleen clustered with monocytes, while skeletal muscle and heart muscle grouped together and were distinct from smooth muscle. Although the PCA and gene expression tree correlated with the broad functional features of the cells and tissues, the clustering did not strictly follow germ layer origin. In agreement, recent data demonstrate that the regulatory DNA landscape (epigenome), but not gene expression (transcriptome), correlate with cell lineage relationships, including germ layer origin.^23–25^ Thus, for any given cell type, e.g., a neuron, epigenetic marks reflect both the prior (e.g., state in the germ layer and derived cell lineages) and present regulatory landscapes.^24^

### Differential gene expression of cell and tissue groups

**Figure 2:**
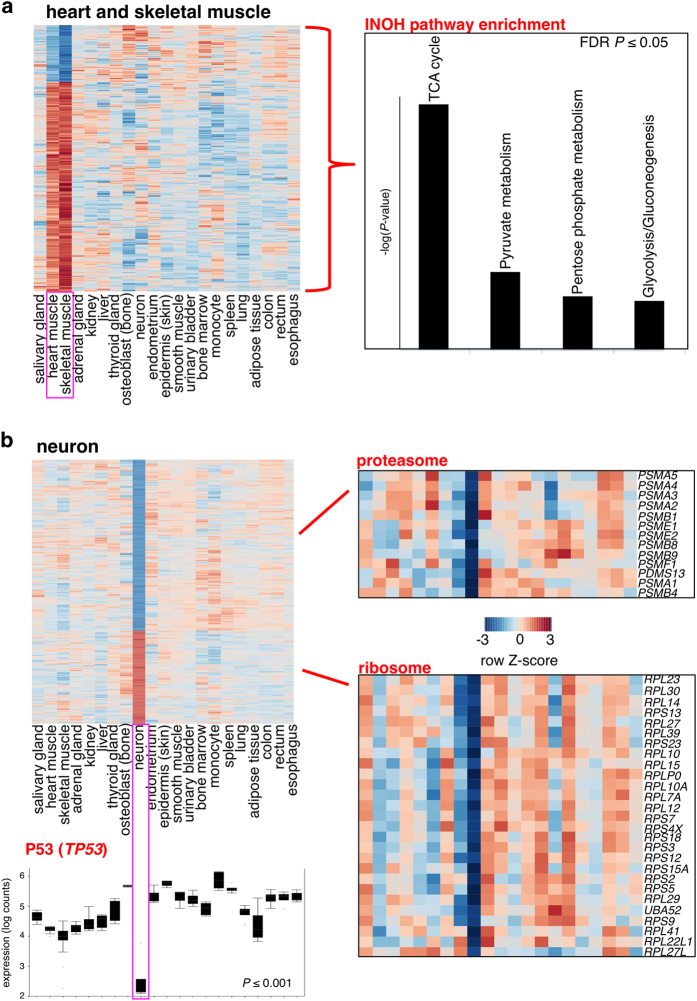
Genes differentially expressed between selected cell lineages and tissues. (**a**) Heart and skeletal muscle. Left panel, heat map of genes differentially expressed in heart and skeletal muscle relative to the other cells and tissues (standardized expression level; red: high expression; blue: low expression). Right panel, significantly enriched (FDR *P*⩽0.05) pathways. (**b**) Neuron. Left panel, heat map of genes differentially expressed in neurons, colored and annotated as in **a**. Right panel, significantly enriched (FDR *P*⩽0.05) pathways ‘ribosome’ and ‘proteasome’. FDR P denotes false discovery rate-adjusted *P* value. Lower panel, box plot showing p53 (*TP53*) expression. Error bars indicate s.e.m.

We selected several lineage groups and individual cells and tissues and compared each of them against all other cells and tissues in order to identify associated specific expression patterns (Supplementary Tables S3 and S4; Supplementary Results and Discussion). The results for heart (muscle), thyroid gland, hematopoietic tissues and monocytes are presented in Supplementary Figure S2. In heart and skeletal muscle, 455 out of 12,044 genes were differentially expressed (phylogenetic analysis of variance (ANOVA) *P* value ⩽0.01) compared with other cells and tissues (Figure 2a). Approximately 44% of these genes were associated with the tricarboxylic acid (TCA) cycle and respiration, in agreement with the metabolic organization and energy sources of these tissues.^26^

Neurons, which are critical for cognitive and motor functions, have cell lifespans that likely exceed the lifespan of the organism.^7,27^ Comparing neurons to shorter-lived cells and tissues is conceptually similar to comparing gene expression of long-lived mammals to related short-lived species, e.g., examining African mole rats against other rodents.^15^ Accordingly, neurons should possess a gene expression signature associated with low turnover/long lifespan, in addition to the patterns indicative of neuronal function. Out of 12,044 genes 1,438 were differentially expressed in neurons (*P*⩽0.01; Figure 2b; Supplementary Table S3) and gene set enrichment analysis showed enrichment for functions associated with lysosomes, proteasomes, ribosomal proteins and apoptosis (Supplementary Table S3). Neurons presented with reduced expression of 27 ribosomal proteins and multiple 20S proteasome subunit genes (Figure 2b), consistent with distinct protein metabolism required to fine-tune self-renewal and synaptic plasticity.^28^ This group of genes was not correlated with cell and tissue turnover (see section below), suggesting that this expression pattern is unique to long-lived neurons. Reduced protein metabolism, which may be induced by dietary restriction and other interventions, is known to associate with extended lifespan in a number of model organisms.^29,30^ Furthermore, expression of the tumor suppressor p53 (*TP53*) was significantly reduced (*P*⩽0.001) in neurons, where it was expressed at a level 5–30 times lower than that in the other cells and tissues (Figure 2b). Reduced p53 expression is associated with a concomitant reduction in cell cycle-related proteins in neurons following their terminal differentiation from neuroblasts.^31^

### Gene expression patterns of cell and tissue turnover

**Figure 3:**
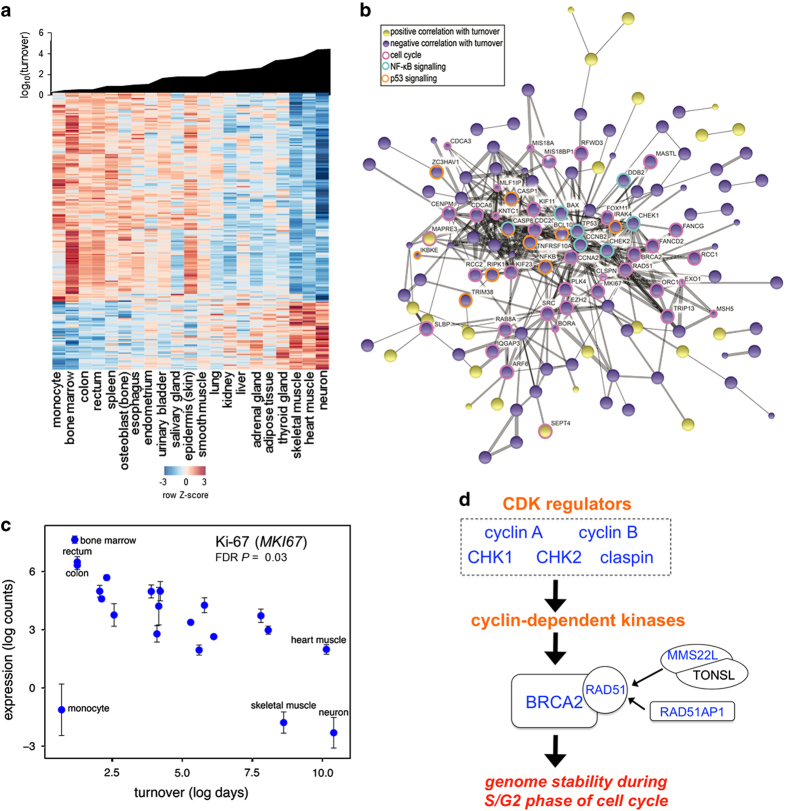
Overview of genes correlating with cellular turnover. (**a**) Heat map analysis. Upper panel, cellular turnover of the cells and tissues (in ascending order). Lower panel, heat map displaying gene expression of genes correlating with turnover (standardized expression levels; red: high expression; blue: low expression). (**b**) Network analysis. Protein–protein interaction network of genes correlating with turnover. The interaction network was created by interrogating the STRING database (evidence view). Lines (strings) indicate protein interactions. Proteins without interacting partners were omitted. Selected pathways are indicated by colored rings. (**c**) Expression of the cell proliferation marker Ki-67 (*MKI67*) correlates negatively with turnover. Error bars indicate standard error of the mean. FDR *P* denotes false discovery rate *P* value. (**d**) Genes associated with RAD51-mediated genome stability control in the replication phase of the cell cycle. Names highlighted in blue showed negative correlation with turnover. CDK: cyclin-dependent kinase; *CCNA2*: cyclin A2; *CCNB2*: cyclin B2; *CHEK1*: checkpoint kinase 1, CHK1; *CHEK2*: checkpoint kinase 2, CHK2; *CLSPN*: claspin; *BRCA2*: breast cancer 2, early onset; *RAD51*: RAD51 recombinase; *RAD51AP1*: RAD51 associated protein 1; *MMS22L*: MMS22-like, DNA repair protein; *TONSL*: tonsoku-like, DNA repair protein.

We identified genes whose expression correlated with cell and tissue turnover. Available turnover times for a number of tissues and cell types (in days)^3^ were supplemented with estimates from the literature and used as a *bona fide* measure of lifespan (‘lifespan trait’). We applied generalized least squares regression,^32^ tested different evolutionary models and selected the best fit model by maximum likelihood (see Extended Experimental Procedures). Two hundred eight out of 12,044 genes showed significant correlation with turnover at a false discovery rate (*Q*-value) of 0.05, with 75% (155 genes) in negative correlation and 25% (53 genes) in positive correlation (Supplementary Table S5). Notable genes with a positive correlation included the complex *SNRPN-SNURF* locus, which gives rise to a number of proteins and short non-coding RNAs (Supplementary Figure S3; Supplementary Results and Discussion). We visualized the protein–protein interaction network represented by these 208 genes, revealing significant enrichment (FDR *P*⩽0.05) for genes involved in cell cycle, immune signaling (NF-κB) and p53 signaling (Figure 3a,b and Supplementary Tables S6 and S7). In our data set, hematopoietic tissues (bone marrow and spleen) and monocytes constituted the samples with the shortest turnover. Removal of these data points in the regression analysis retained the ‘turnover signature’, with the overlapping gene set comprising critical cell cycle and apoptosis associated genes, such as *CHEK1*, *CHEK2*, *MKI67*, *FOXM1, TP53* and *BCL10*, while a correlation with immune signaling-associated genes was lost (Table 2 and Supplementary Table S8).

### Negative correlation between cell cycle and associated genome integrity pathways and cell and tissue turnover

Gene ontology, KEGG pathway analysis and manual interrogation of genes correlating with turnover revealed that 30–40% of these (Supplementary Tables S5 and S8) have roles in the cell cycle, a highly complex multi-step process. They spanned all phases of the cell cycle, with the majority of the genes showing decreased expression associated with increased turnover. As expected, the classic DNA replication marker, Ki-67 (*MKI67*), which is only expressed during the cell cycle,^33^ was present at high levels in rapidly proliferating tissues such as bone marrow, rectum and colon. It was expressed at very low levels in monocytes (the majority of which do not proliferate) and in post-mitotic skeletal muscle and neurons (most of which are incapable of cell division; Figure 3c). Ki-67 was expressed at a moderate level in heart muscle, which can proliferate at a limited rate,^9^ and where Ki-67 may promote postnatal cardiac remodeling.^34^

A negative correlation between the cell cycle machinery and cell and tissue turnover is not surprising, as proliferative homeostasis, a balance between cell growth and death, is essential for normal turnover (as occurs in epidermal cells, for example). In contrast, terminally differentiated cells comprising heart muscle, skeletal muscle and neurons, have permanently or largely exited the cell cycle. They are, therefore, expected to express low levels of genes related to cell cycle checkpoints and the maintenance of replication fidelity. We noted that a number of genes essential for genome stability during the premitotic phase (G2), a process tightly linked to tumor development,^35^ were negatively correlated with turnover (Figure 3d). This included regulators of cyclin-dependent kinases, such as cyclin A (*CCNA2)* and B (*CCN2B)*, checkpoint kinase 1 (*CHEK1*) and its regulator claspin (*CLSPN*), and *CHEK2*, which together with breast cancer 2 early onset (*BRCA2*), RAD51 recombinase (*RAD51*), its enhancer RAD51AP1 (*RAD51AP1*), and MMS22-like DNA repair protein (*MMS22L*) promote genome stability.^35^ These data support recent work showing that RAD51 and BRCA2 are major facilitators of genome integrity in proliferating cells.^35–37^ Genes in the p53 pathway can halt progression of the cell cycle (induce senescence), or inhibit apoptosis in proliferating cells, and blocking apoptosis is crucial for the survival of differentiated post-mitotic cells.^38^ In agreement with a reduction in the involvement of cell cycle in longer-lived cells and tissues, multiple p53 pathway associated genes, including p53 itself (*TP53*), were negatively correlated with turnover (Supplementary Figure S4; Supplementary Tables S5 and S8). They encompassed several checkpoint kinases (*CHEK1* and *CHEK2)*, BCL2-associated X protein (*BAX*), which activates apoptosis, and ASC/TMS1 (*PYCARD*), which when downregulated inhibits BAX translocation to mitochondria.

To further investigate the connection between gene expression and turnover, we turned to the library of integrated network-based cellular signatures (LINCS) to identify perturbations (gene overexpression and knockdown) that produce a similar overall gene expression profile to our regression analysis. We found that the most similar profiles came from genes associated with cell cycle control and associated DNA repair (Supplementary Table S11). This included overexpression of the major cell cycle inhibitors p18 (also known as INK4C; *CDKN2C*), p21 (*CDKN1A*) and p27 (*CDKN1B*), in agreement with the negative correlation between downstream genes and turnover in our data set.

### No correlation between oxidative phosphorylation gene expression and cell and tissue turnover

**Figure 4:**
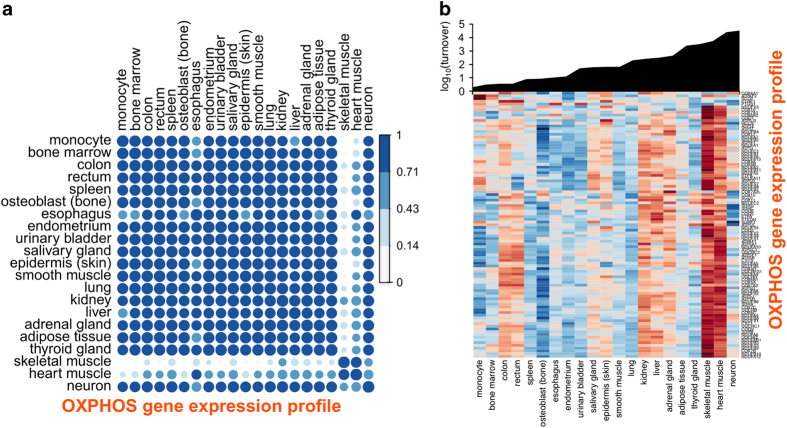
Expression of nuclear-encoded oxidative phosphorylation (OXPHOS) genes in 21 human somatic cells and tissues. (**a**) Pairwise correlation matrix of 97 OXPHOS genes. Darker blue shading indicating stronger positive correlation. Blue corresponds to a correlation of ~1 and white to ~0. (**b**) Upper panel, cellular turnover of the cells and tissues (in ascending order). Lower panel, heat map displaying gene expression of individual OXPHOS genes. Scaled log_2_ transformed normalized counts (*Z*-score) are plotted in blue–red color, with red indicating high expression and blue indicating low expression.

It is clear that mitochondrial dysfunction is a hallmark of aging. Properly functioning mitochondria are essential for energy production and cell survival, and hence, are crucial for longevity and resistance to age-related disease.^39^ We calculated pair-wise correlations of the expression of 97 nuclear-encoded genes associated with oxidative phosphorylation (OXPHOS)^40^ across the data set, revealing a strong correlation across all samples with the exception of heart muscle and skeletal muscle (Figure 4a). As shown in Figure 4b, expression of individual OXPHOS genes varied by tissue, in agreement with a previous microarray study,^40^ but there was no overall correlation with cell and tissue turnover.

## Discussion

Several interrelated evolutionary and mechanistic theories have been proposed that provide insights into the evolution of lifespan and suggest the involvement of a large armamentarium of genes.^41,42^ In this study, we employed gene expression data from 21 somatic cell types and tissues and sought to identify genes and pathways associated with cell and tissue turnover, to our knowledge the current best estimate of cellular lifespan,^1^ in one of the longest-lived mammals, human. We first tested for differential expression in selected groups of cells and tissues, revealing expression patterns that fit well with expected biological functions, including the TCA cycle and respiration in cardiac and skeletal muscle; immune function genes in bone marrow, monocyte and spleen, and reduced protein metabolism in neurons.

The major insights of our study centered on the relationship between gene expression and cell and tissue turnover. Multiple genes taking part in the energetically expensive cell cycle and associated repair (genome stability) were negatively correlated with turnover. By interrogating the Broad Institute’s Library of Integrated Cellular Signatures resource, we found that very similar gene expression patterns can be achieved by targeted overexpression and knockdown of single genes, suggesting that gene expression patterns associated with cell and tissue turnover, and by proxy cell lifespan, may in principle be achieved by genetic, pharmacological and perhaps dietary interventions. The observation that turnover negatively correlates with cell cycle genes may seem tautological. Indeed, one would expect tissue or cell populations with high turnover to exhibit more cells in the cell cycle and, consequently, high expression of cell cycle and associated genes. However, it is recognized that the rate of aging and longevity is indeed fine tuned by the balance of cell division and death (i.e., cell turnover).^2^ Interestingly, a recent study examined the transcriptional response to long-term calorie restriction in humans, revealing that calorie restriction shifts cellular metabolism of skeletal muscle from proliferation to maintenance and repair.^43^ The concomitant reduction in cell cycle gene expression presumably results in a ‘younger’ transcriptional signature that contributes to the lifespan-extending properties of calorie restriction.

Expression of p53 (*TP53*), often referred to as the ‘guardian of the genome’, was negatively correlated with cell and tissue turnover; with particularly low levels of expression in long-lived neurons. Evidence is emerging that p53 has an evolutionarily ancient lifespan-regulating function, in addition to its role as tumor suppressor. It is appreciated that p53 promotes organismal longevity by preventing survival of abnormal cells; however, several investigators have speculated that p53 protects against cancer in proliferating cells at the cost of accelerated aging.^3,44,45^ Thus, in non-dividing cells p53 may, in effect, reduce lifespan. It was found that suppression of *TP53* orthologs in animal models such as the mouse and fruit fly can extend organismal lifespan (reviewed in ref. 46). Dominant-negative *Drosophila* p53 (*Dmp53*) significantly extends organismal lifespan when expressed in adult neurons, but not other tissues (such as muscle) via insulin/insulin-like signaling (IIS) and TOR (target of rapamycin) pathways.^47,48^ Several p53 (*TP53*) retrogenes have been recently reported in the elephant genome,^49^ however, while elephant lymphocytes and fibroblasts show an increased response to DNA damage compared with human cells,^49^ it is currently not known how many of these retrogenes are actively translated and exhibit p53 function, nor whether the expression of p53 is appropriately activated and/or elevated in all elephant cells. Thus, the elephant’s resistance to cancer may stem from other mechanisms and it would be of interest to examine the expression of *TP53* in elephant neurons and other cell types. Interestingly, the cancer-resistant long-lived blind mole rat, *Spalax*, has evolved an enhanced necrotic and reduced apoptotic defense (via a dominant negative form of p53) against cancer, possibly to adapt to an oxygen-poor underground environment, which would normally result in extensive p53-mediated cell death.^50,51^ Whether *Spalax* p53 contributes to the longevity of this cancer-resistant rodent is not known, but is an exciting possibility under the hypothesis that reduced p53 activity exerts beneficial effects on cellular lifespan if tumor formation is avoided. Taken together, we speculate that very low levels of endogenous p53 contribute to the exceptional lifespan of cells and tissues with low turnover, such as neuron, heart muscle and skeletal muscle, and perhaps also organismal longevity.

It is now appreciated that expression of OXPHOS genes decreases with age in diverse cells and tissues in species ranging from nematode to human.^39^ However, the link between mitochondrial homeostasis and lifespan is currently enigmatic.^39^ Interestingly, lifespans of different strains of the single-celled yeast is associated with upregulation of OXPHOS genes.^52^ In our data set of 21 human cells and tissues, expression of individual OXPHOS genes, and more generally metabolic genes, did not correlate with cell turnover. Thus, we propose that while cells and tissues share a gene expression signature manifested as reduced mitochondrial function with age, the overall integrity of mitochondria in long-lived human cell types is achieved by distinct gene expression strategies.

Our study has several limitations. Future studies should attempt to more accurately determine the turnover of cells and cell populations (tissues) in the body, and sequence the transcriptomes of additional long-lived post-mitotic cells, such as osteocytes.^53^ Single-cell RNA sequencing is rapidly evolving^54^ and would greatly advance the study of cell turnover, especially as it would avoid the analysis of organs that are composed of heterogeneous cell types. Multiple cells and tissues from the same individual are also becoming available.^55^ Finally, the contribution of epigenetics^56,57^ and long-lived proteins^58,59^ to cell and tissue turnover is largely unknown but likely important. Owing to a lack of a matching set of samples and limited number of biological replicates, we did not interrogate other species, such as the mouse, for turnover-associated genes. It is currently unknown whether individual genes identified through our procedure would overlap among species,^60^ but is an important question for future studies. Although some common features may be observed, human cell types may also harbor signatures quite distinct from other animals, including other primates, since humans are one of the most exceptionally long-lived species.^61^

Overall, our analysis, employing cellular turnover, as a proxy of lifespan, is a first step to a molecular understanding of cell and tissue longevity. We reveal a gene signature of exceptionally long-lived post-mitotic neurons, and genes and pathways that correlate with turnover across 21 somatic cells and tissues. The data suggest that human cell lineages utilize both common and lineage-specific strategies to alter their lifespan. This new perspective should provide further impetus to the study of the lifespan trait (longevity) and the aging process.

## Materials and methods

See Supplementary Information for detailed methods.

### Biological samples

Our analysis was restricted to 21 adult somatic cells and tissues with more than three biological replicates and *bona fide* lifespan estimates (cell turnover in days) derived from a recent comprehensive review^3^ and the additional data collected through primary literature searches (see Supplementary Experimental Procedures).

### Transcriptome data relationship inference

The relationship of publicly available transcriptomes (RNA-seq data) from the 21 cells and tissues was investigated by principal component analysis and a gene expression tree (see Supplementary Experimental Procedures).

### Identification of genes differentially expressed between cell and tissue groups

Differentially expressed genes in a particular group (e.g., heart and skeletal muscle) were identified by ANOVA, taking into account the hierarchical relationship between samples in our data set (tissue autocorrelation by ‘phylogenetic ANOVA’). See Supplementary Experimental Procedures for further details.

### Identification of genes correlating with cell and tissue turnover

To identify genes correlating with cell turnover (a *bona fide* lifespan estimate) we employed generalized least squares regression (Supplementary Experimental Procedures).

## Figures and Tables

**Table 1:** Summary of human cells and tissues used in the study

*Tissue/cell type*	*Germ layer*	*Estimated turnover (days)*
Adipose tissue	Mesoderm	2,448
Adrenal gland	Ectoderm	455
Bone marrow	Mesoderm	3.2
(CD14^+^) monocytes	Mesoderm	2
Colon	Endoderm	3.5
Endometrium	Mesoderm	13
Esophagus	Endoderm	10
Heart muscle	Mesoderm	25,300
Keratinocytes (skin epidermis)	Ectoderm	64
Kidney	Mesoderm	270
Liver	Endoderm	327
Lung	Endoderm	200
Neuron (neocortex)	Ectoderm	32,850
Osteoblasts (bone)	Mesoderm	8.3
Rectum	Endoderm	3.5
Salivary gland	Ectoderm	60
Skeletal muscle	Mesoderm	5,510
Smooth muscle	Mesoderm	67.5
Spleen	Mesoderm	7.8
Thyroid gland	Endoderm	3,180
Urinary bladder	Endoderm	49

See Supplementary Table S1 for further details.

**Table 2:** Overlap of genes correlating with turnover before and after removal of immune system-associated cells and tissues (monocytes, bone marrow and spleen)

*Gene*	*Description*	*Correlation with cell turnover*	*Function(s)*
*BCL10*	B-cell CLL/lymphoma 10	Negative	Apoptosis
*BRCA2*	Breast cancer 2, early onset	Negative	Cell cycle
*CCDC92*	Coiled-coil domain containing 92/limkain beta-2	Positive	—
*CCNB2*	Cyclin B2	Negative	Cell cycle
*CDC42*	Cell division cycle 42	Negative	Cell cycle
*CDCA3*	Cell division cycle associated 3	Negative	Cell cycle
*CDCA8*	Cell division cycle associated 8	Negative	Cell cycle
*CENPW*	Centromere protein W	Negative	Cell cycle
*CHEK1*	Checkpoint kinase 1	Negative	Cell cycle
*CHEK2*	Checkpoint kinase 2	Negative	Cell cycle
*CRELD1*	Cysteine-rich with EGF-like domains 1	Positive	Putative cell adhesion molecule
*CRY2*	Cryptochrome circadian clock 2	Positive	Core circadian clock gene
*DDB2*	Damage-specific DNA binding protein 2, 48 kDa	Negative	DNA repair
*EXO1*	Exonuclease 1	Negative	DNA repair
*FANCD2*	Fanconi anemia, complementation group D2	Negative	Cell cycle
*FOXM1*	Forkhead box M1	Negative	Cell cycle
*HEY1*	Hes-related family bHLH transcription factor with YRPW motif 1	Positive	Transcription factor
*HNRNPF*	Heterogeneous nuclear ribonucleoprotein F	Negative	mRNA stability and transport
*KIF11*	Kinesin family member 11	Negative	Cell cycle
*KIF23*	Kinesin family member 23	Negative	Cell cycle
*MKI67*	Marker of proliferation Ki-67	Negative	Cell cycle
*MSH5*	mutS homolog 5	Negative	DNA repair
*NADSYN1*	NAD synthetase 1	Negative	Redox reaction coenzyme, precursor for cell signaling molecules, and substrate for protein post-translational modifications
*NCAPG*	Non-SMC condensin I complex, subunit G	Negative	Cell cycle
*NCAPH*	Non-SMC condensin I complex, subunit H	Negative	Cell cycle
*NUF2*	NUF2, NDC80 kinetochore complex component	Negative	Cell cycle
*ORC1*	Origin recognition complex, subunit 1	Negative	Cell cycle
*PARPBP*	PARP1 binding protein	Negative	Cell cycle
*PLK4*	Polo-like kinase 4	Negative	Cell cycle
*RCC1*	Regulator of chromosome condensation 1	Negative	Cell cycle
*SAMD9*	Sterile alpha motif domain containing 9	Negative	Apoptosis
*SNRPN*	Small nuclear ribonucleoprotein polypeptide N	Positive	Complex SNURF-SNRPN locus: mRNA processing, short non-coding RNA precursor
*STK26*	Serine/threonine protein kinase 26	Negative	Apoptosis
*STK38*	Serine/threonine kinase 38	Negative	Cell cycle and apoptosis
*TP53*	Tumor protein p53	Negative	Apoptosis
*ZWINT*	ZW10 interacting kinetochore protein	Negative	Cell cycle

Abbreviation: mRNA, messenger RNA.
