# Supplementary material for: Gene expression signatures of human cell and tissue longevity
Source: NPJ Aging Mech Dis. 2016 Jul 7;2:16014–. doi: 10.1038/npjamd.2016.14 (PMC5514998; doi:10.1038/npjamd.2016.14)
Supplement: Supplementary Information [file npjamd201614-s1.doc]

**Gene expression signatures of human cell and tissue longevity**

Inge Seim^1^, Siming Ma^1^, and Vadim N. Gladyshev^1^*

^1^ Division of Genetics, Department of Medicine, Brigham and Women’s Hospital and Harvard Medical School, Boston, MA, 02115, USA

* Corresponding author: Division of Genetics, Department of Medicine, Brigham & Women's Hospital and Harvard Medical School, New Research Building, Room 435, 77 Avenue Louis Pasteur, Boston, MA 02115 USA. Phone: (617) 525-5122. E-mail: vgladyshev@rics.bwh.harvard.edu

Supplementary Information

**SUPPLEMENTARY FIGURE 1**


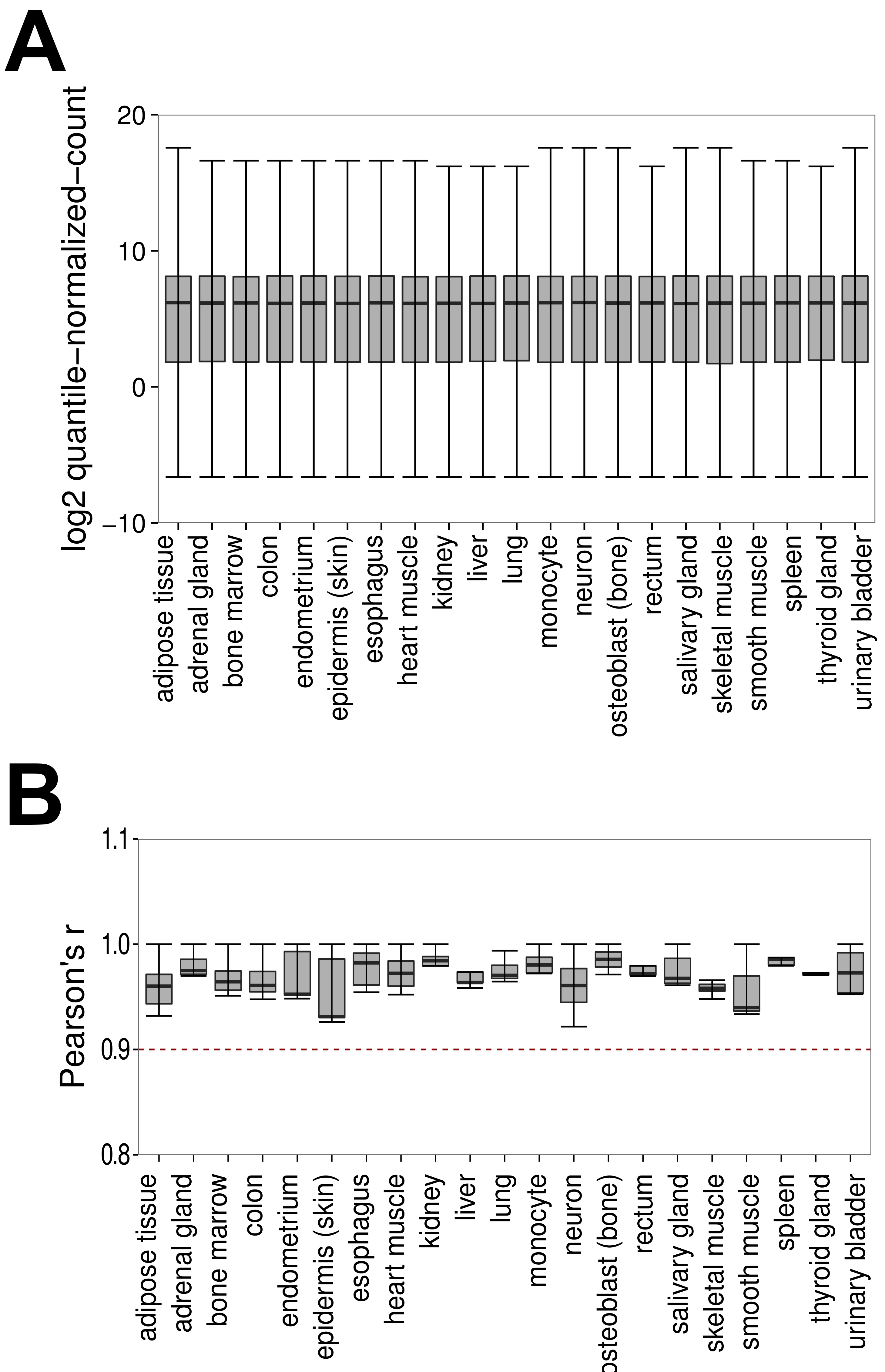


**Supplementary Figure 1. RNA-seq quality assessment.**

**(A)** Boxplot of quantile-normalized RNA-seq counts.

**(B)** Boxplot of correlation coefficients (Pearson's r) between biological replicates. A correlation coefficient between 0.9 and 1.0 indicate samples that can be considered highly correlated.

**SUPPLEMENTARY FIGURE 2**

**
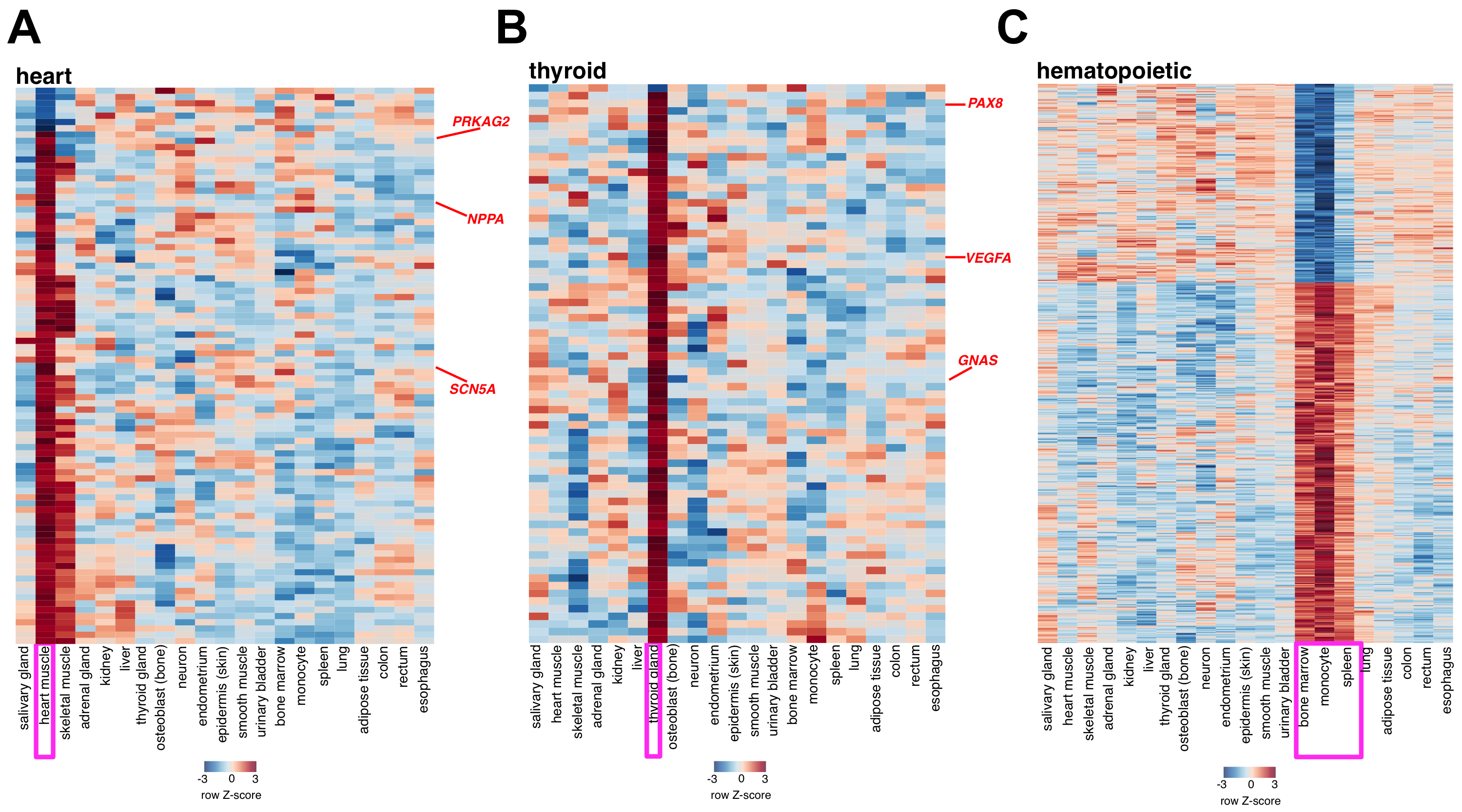
**

**Supplementary Figure 2. Genes with high expression divergence between selected cells and tissues, as measured by Phylogenetic ANOVA.**

**(A)** Heart.

**(B)** Thyroid (gland).

**(C)** Hematopoietic; spleen, bone marrow and monocyte.

Phylogenetic ANOVA was performed by comparing the highlighted ‘lineage’ to all other samples, with a phylogeny-adjusted *P*-value ≤ 0.01 considered significant. Scaled log_2_ transformed normalized read counts (denoted as the row Z-score) are plotted in blue–red color, with red indicating high expression and blue indicating low expression.

**
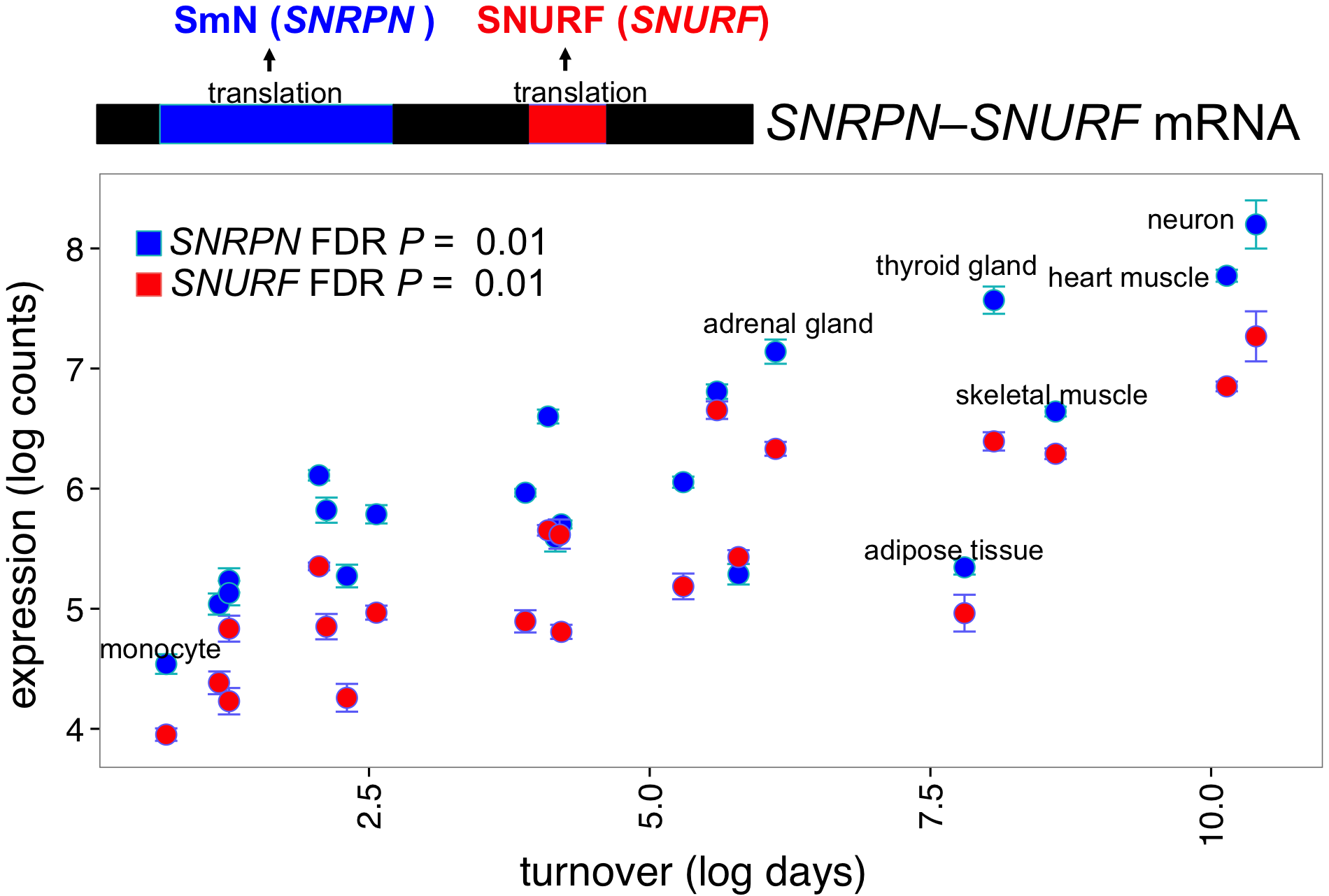
**

**Supplementary Figure 3. The complex *SNRPN*-*SNURF* locus positively correlates with cellular turnover.**

A bicistronic *SNRPN*-*SNURF* mRNA gives rise to the proteins SNRPN (small nuclear ribonucleoprotein polypeptide N; SmN) and SNURF (SNRPN upstream reading frame). *SNRPN* and *SNURF* coding exon sequences and scatterplot points are shown as blue and red, respectively. Error bars indicate standard error of the mean. FDR *P* denotes false discovery rate *P*-value.

**
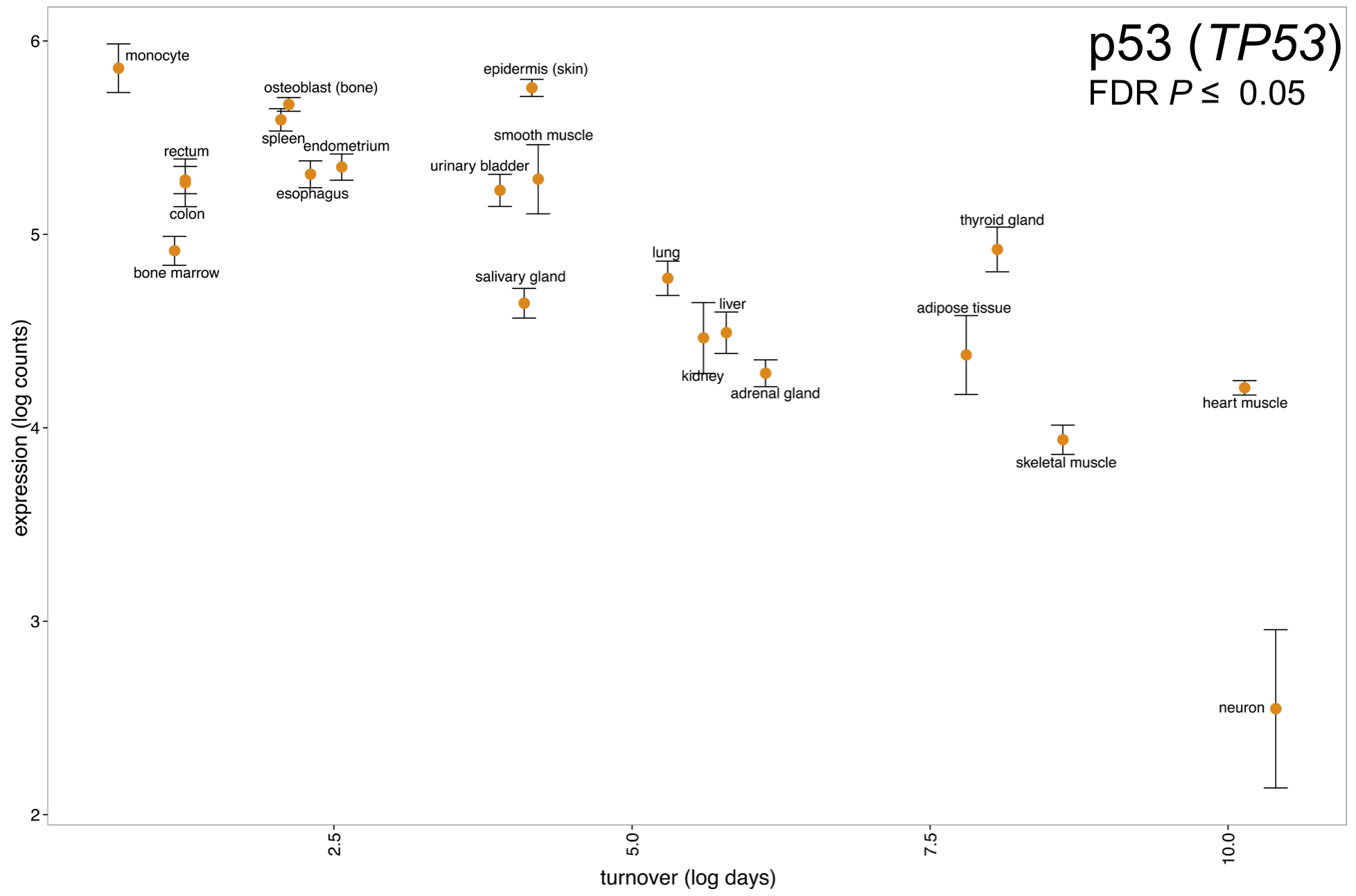
**

**Supplementary Figure 4. The ‘guardian of the genome’, p53 (*TP53*), negatively correlates with cellular turnover.**

Error bars indicate standard error of the mean. FDR *P* denotes false discovery rate *P*-value.

**Supplementary Table 1. Detailed information of RNA-seq samples.**

Ostensible germ layer origins were derived from Vickaryous and Hall[^1^](#_ENREF_1). NA: Not Available; HPA: Human Protein Atlas.

| **Tissue/cell type** | **Germ layer** | **Estimated**  **turnover (days)** | **Literature**  **source(s)** | **Number**  **of samples** | **Mean age of human donors (years)** |
| --- | --- | --- | --- | --- | --- |
| Adipose tissue | mesoderm | 2,448 | [2-4](#_ENREF_2) | 7 | 63 |
| Adrenal gland | ectoderm | 455 | [5](#_ENREF_5) | 6 | 54 |
| Bone marrow | mesoderm | 3.2 | [6](#_ENREF_6) | 8 | 47 |
| (CD14^+^) monocyte | mesoderm | 2 |  | 6 | NA |
| Colon | endoderm | 3.5 |  | 8 | 65 |
| Endometrium | mesoderm | 13 |  | 9 | 55 |
| Esophagus | endoderm | 10 | [13](#_ENREF_13) | 6 | 61 |
| Heart muscle | mesoderm | 25,300 |  | 9 | 53 |
| Keratinocyte (skin epidermis) | ectoderm | 64 | [16](#_ENREF_16) | 5 | NA |
| Kidney | mesoderm | 270 | [5](#_ENREF_5) | 4 | 57 |
| Liver | endoderm | 327 | [17](#_ENREF_17) | 5 | 63 |
| Lung | endoderm | 200 | [18](#_ENREF_18) | 8 | 69 |
| Neuron (neocortex) | ectoderm | 32,850 |  | 4 | 66 |
| Osteoblast (bone) | mesoderm | 8.3 |  | 2 | NA |
| Rectum | endoderm | 3.5 |  | 4 | 70 |
| Salivary gland | ectoderm | 60 | [23](#_ENREF_23) | 6 | 50 |
| Skeletal muscle | mesoderm | 5,510 | [19](#_ENREF_19) | 32 | HPA(6): NA  [^24^](#_ENREF_24)(26): 27 |
| Smooth muscle | mesoderm | 67.5 |  | 3 | 54 |
| Spleen | mesoderm | 7.8 | [27](#_ENREF_27) | 5 | 29 |
| Thyroid gland | endoderm | 3,180 | [28](#_ENREF_28) | 9 | 45 |
| Urinary bladder | endoderm | 49 | [29](#_ENREF_29) | 6 | 77 |

**Supplementary Table 2. Summary of RNA-sequencing data.**

Aligned paired-reads in TopHat, Pearson coefficients of biological replicates and literature sources of the samples are listed.

| **sample name** | **sample type** | **aligned paired reads** | **Pearson correlation of biological replicate** | **reference(s)** |
| --- | --- | --- | --- | --- |
| ERR315332 | adipose tissue | 13,679,690 | 1.00 |  |
| ERR315342 | adipose tissue | 15,718,516 | 0.97 |  |
| ERR315343 | adipose tissue | 2,138,967 | 0.95 |  |
| ERR315378 | adipose tissue | 7,371,560 | 0.97 |  |
| ERR315431 | adipose tissue | 2,508,174 | 0.96 |  |
| ERR579122 | adipose tissue | 31,982,215 | 0.93 |  |
| ERR579146 | adipose tissue | 38,163,297 | 0.93 |  |
| ERR315335 | adrenal gland | 4,699,590 | 1.00 |  |
| ERR315385 | adrenal gland | 5,238,333 | 0.97 |  |
| ERR315392 | adrenal gland | 4,501,896 | 0.97 |  |
| ERR315417 | adrenal gland | 7,066,673 | 0.97 |  |
| ERR315450 | adrenal gland | 6,093,356 | 0.98 |  |
| ERR315452 | adrenal gland | 6,346,397 | 0.99 |  |
| ERR315333 | bone marrow | 13,209,582 | 1.00 |  |
| ERR315395 | bone marrow | 13,025,511 | 0.99 |  |
| ERR315396 | bone marrow | 12,871,598 | 0.97 |  |
| ERR315404 | bone marrow | 13,741,727 | 0.96 |  |
| ERR315406 | bone marrow | 13,906,375 | 0.96 |  |
| ERR315425 | bone marrow | 11,649,721 | 0.95 |  |
| ERR315469 | bone marrow | 11,879,854 | 0.95 |  |
| ERR315486 | bone marrow | 12,707,596 | 0.97 |  |
| ERR315348 | colon | 9,856,467 | 1.00 |  |
| ERR315357 | colon | 28,063,977 | 0.96 |  |
| ERR315400 | colon | 15,405,148 | 0.97 |  |
| ERR315403 | colon | 3,977,193 | 0.96 |  |
| ERR315462 | colon | 8,956,738 | 0.96 |  |
| ERR315484 | colon | 19,398,532 | 0.98 |  |
| ERR579129 | colon | 35,017,647 | 0.95 |  |
| ERR579148 | colon | 43,462,253 | 0.95 |  |
| ERR315361 | endometrium | 6,042,492 | 1.00 |  |
| ERR315368 | endometrium | 5,465,669 | 0.95 |  |
| ERR315386 | endometrium | 4,179,293 | 0.95 |  |
| ERR315433 | endometrium | 5,475,801 | 0.95 |  |
| ERR315438 | endometrium | 4,185,955 | 0.95 |  |
| ERR315490 | endometrium | 6,062,592 | 0.99 |  |
| ERR315495 | endometrium | 10,811,413 | 0.99 |  |
| ERR579123 | endometrium | 33,679,045 | 0.96 |  |
| ERR579138 | endometrium | 35,419,710 | 0.95 |  |
| ENCSR000CPL-keratinocyte-5-1 | epidermis (skin) | 112,816,849 | 1.00 | [^32^](#_ENREF_32) |
| ENCSR000EYT-keratinocyte-1-1 | epidermis (skin) | 12,974,589 | 0.93 | [^32^](#_ENREF_32) |
| ENCSR000EYT-keratinocyte-1-2 | epidermis (skin) | 12,299,960 | 0.93 | [^32^](#_ENREF_32) |
| ENCSR000EYT-keratinocyte-1-3 | epidermis (skin) | 8,015,234 | 0.93 | [^32^](#_ENREF_32) |
| ENCSR000EYT-keratinocyte-2-1 | epidermis (skin) | 26,677,926 | 0.99 | [^32^](#_ENREF_32) |
| ERR315362 | esophagus | 9,306,826 | 1.00 |  |
| ERR315398 | esophagus | 11,071,355 | 0.98 |  |
| ERR315411 | esophagus | 9,884,140 | 0.99 |  |
| ERR315434 | esophagus | 7,662,469 | 0.95 |  |
| ERR315472 | esophagus | 10,432,584 | 0.98 |  |
| ERR315489 | esophagus | 8,142,589 | 0.95 |  |
| ERR315328 | heart muscle | 10,360,590 | 1.00 |  |
| ERR315331 | heart muscle | 11,482,236 | 0.96 |  |
| ERR315356 | heart muscle | 13,626,629 | 0.95 |  |
| ERR315367 | heart muscle | 11,643,965 | 0.96 |  |
| ERR315384 | heart muscle | 13,654,400 | 0.97 |  |
| ERR315389 | heart muscle | 6,095,482 | 0.98 |  |
| ERR315413 | heart muscle | 13,626,741 | 0.97 |  |
| ERR315430 | heart muscle | 13,358,866 | 0.95 |  |
| ERR315435 | heart muscle | 6,076,168 | 0.98 |  |
| ERR315383 | kidney | 11,022,817 | 1.00 |  |
| ERR315443 | kidney | 14,801,166 | 0.98 |  |
| ERR315468 | kidney | 7,389,319 | 0.97 |  |
| ERR315494 | kidney | 10,068,226 | 0.98 |  |
| ERR315327 | liver | 4,709,463 | 1.00 |  |
| ERR315394 | liver | 4,685,285 | 0.97 |  |
| ERR315414 | liver | 13,756,011 | 0.96 |  |
| ERR315451 | liver | 11,253,297 | 0.96 |  |
| ERR315463 | liver | 11,429,118 | 0.96 |  |
| ERR315326 | lung | 4,967,444 | 1.00 |  |
| ERR315341 | lung | 20,476,728 | 0.97 |  |
| ERR315346 | lung | 17,083,332 | 0.97 |  |
| ERR315353 | lung | 5,866,789 | 0.96 |  |
| ERR315424 | lung | 3,717,500 | 0.98 |  |
| ERR315439 | lung | 3,229,871 | 0.97 |  |
| ERR315444 | lung | 3,288,873 | 0.97 |  |
| ERR315487 | lung | 7,892,387 | 0.99 |  |
| ENCSR000CUC-CD14-positive-monocyte-1-1 | monocyte | 16,998,339 | 1.00 | [^32^](#_ENREF_32) |
| ENCSR000CUC-CD14-positive-monocyte-1-2 | monocyte | 17,581,190 | 0.99 | [^32^](#_ENREF_32) |
| ENCSR000CUC-CD14-positive-monocyte-1-3 | monocyte | 17,323,513 | 0.99 | [^32^](#_ENREF_32) |
| ENCSR000CUC-CD14-positive-monocyte-2-1 | monocyte | 17,329,200 | 0.97 | [^32^](#_ENREF_32) |
| ENCSR000CUC-CD14-positive-monocyte-2-2 | monocyte | 17,810,277 | 0.97 | [^32^](#_ENREF_32) |
| ENCSR000CUC-CD14-positive-monocyte-2-3 | monocyte | 17,888,947 | 0.97 | [^32^](#_ENREF_32) |
| SRR1206036 | neuron | 28,582,237 | 1.00 | [^20^](#_ENREF_20) |
| SRR1206037 | neuron | 31,047,471 | 0.97 | [^20^](#_ENREF_20) |
| SRR1206038 | neuron | 36,924,399 | 0.92 | [^20^](#_ENREF_20) |
| SRR1206039 | neuron | 24,776,033 | 0.95 | [^20^](#_ENREF_20) |
| ENCSR000CUF-osteoblast-1-1 | osteoblast (bone) | 156,890,689 | 1.00 | [^32^](#_ENREF_32) |
| ENCSR000CUF-osteoblast-2-1 | osteoblast (bone) | 151,690,295 | 0.97 | [^32^](#_ENREF_32) |
| ERR579127 | rectum | 33,859,853 | 1.00 |  |
| ERR579140 | rectum | 37,902,377 | 0.97 |  |
| ERR579147 | rectum | 32,008,071 | 0.97 |  |
| ERR579151 | rectum | 38,609,843 | 0.97 |  |
| ERR315325 | salivary gland | 10,435,059 | 1.00 |  |
| ERR315382 | salivary gland | 10,607,164 | 0.99 |  |
| ERR315418 | salivary gland | 7,043,950 | 0.96 |  |
| ERR315420 | salivary gland | 10,852,477 | 0.97 |  |
| ERR315449 | salivary gland | 6,970,847 | 0.96 |  |
| ERR315459 | salivary gland | 11,039,613 | 0.97 |  |
| ERR579130 | skeletal muscle | 8,613,622 | 1.00 |  |
| ERR579141 | skeletal muscle | 8,836,311 | 0.96 |  |
| ERR579142 | skeletal muscle | 8,155,622 | 0.95 |  |
| ERR579143 | skeletal muscle | 4,033,724 | 0.94 |  |
| ERR579149 | skeletal muscle | 9,477,219 | 0.95 |  |
| ERR579152 | skeletal muscle | 7,514,321 | 0.96 |  |
| SRR1424731 | skeletal muscle | 10,383,681 | 0.96 | [^24^](#_ENREF_24) |
| SRR1424732 | skeletal muscle | 11,102,989 | 0.96 | [^24^](#_ENREF_24) |
| SRR1424733 | skeletal muscle | 19,407,539 | 0.96 | [^24^](#_ENREF_24) |
| SRR1424734 | skeletal muscle | 13,419,304 | 0.95 | [^24^](#_ENREF_24) |
| SRR1424735 | skeletal muscle | 21,755,858 | 0.96 | [^24^](#_ENREF_24) |
| SRR1424736 | skeletal muscle | 12,898,986 | 0.96 | [^24^](#_ENREF_24) |
| SRR1424737 | skeletal muscle | 12,333,249 | 0.96 | [^24^](#_ENREF_24) |
| SRR1424738 | skeletal muscle | 9,226,277 | 0.96 | [^24^](#_ENREF_24) |
| SRR1424739 | skeletal muscle | 19,396,953 | 0.97 | [^24^](#_ENREF_24) |
| SRR1424740 | skeletal muscle | 11,778,037 | 0.99 | [^24^](#_ENREF_24) |
| SRR1424741 | skeletal muscle | 6,109,444 | 0.96 | [^24^](#_ENREF_24) |
| SRR1424742 | skeletal muscle | 15,341,805 | 0.96 | [^24^](#_ENREF_24) |
| SRR1424743 | skeletal muscle | 10,867,355 | 0.96 | [^24^](#_ENREF_24) |
| SRR1424744 | skeletal muscle | 14,307,991 | 0.96 | [^24^](#_ENREF_24) |
| SRR1424745 | skeletal muscle | 10,750,775 | 0.96 | [^24^](#_ENREF_24) |
| SRR1424746 | skeletal muscle | 9,331,743 | 0.96 | [^24^](#_ENREF_24) |
| SRR1424747 | skeletal muscle | 9,943,023 | 0.96 | [^24^](#_ENREF_24) |
| SRR1424748 | skeletal muscle | 11,669,684 | 0.96 | [^24^](#_ENREF_24) |
| SRR1424749 | skeletal muscle | 18,352,002 | 0.96 | [^24^](#_ENREF_24) |
| SRR1424750 | skeletal muscle | 13,895,243 | 0.94 | [^24^](#_ENREF_24) |
| SRR1424751 | skeletal muscle | 5,661,423 | 0.95 | [^24^](#_ENREF_24) |
| SRR1424752 | skeletal muscle | 11,773,405 | 0.96 | [^24^](#_ENREF_24) |
| SRR1424753 | skeletal muscle | 17,814,068 | 0.96 | [^24^](#_ENREF_24) |
| SRR1424754 | skeletal muscle | 20,423,449 | 0.96 | [^24^](#_ENREF_24) |
| SRR1424755 | skeletal muscle | 5,618,474 | 0.95 | [^24^](#_ENREF_24) |
| SRR1424756 | skeletal muscle | 4,739,452 | 0.94 | [^24^](#_ENREF_24) |
| ERR579125 | smooth muscle | 31,491,954 | 1.00 |  |
| ERR579131 | smooth muscle | 28,510,726 | 0.93 |  |
| ERR579153 | smooth muscle | 32,073,223 | 0.94 |  |
| ERR315338 | spleen | 18,167,169 | 1.00 |  |
| ERR315405 | spleen | 7,388,796 | 0.98 |  |
| ERR315416 | spleen | 7,191,016 | 0.98 |  |
| ERR315448 | spleen | 18,877,858 | 0.99 |  |
| ERR315473 | spleen | 16,191,328 | 0.99 |  |
| ERR315337 | thyroid gland | 18,756,280 | 1.00 |  |
| ERR315358 | thyroid gland | 13,535,594 | 0.96 |  |
| ERR315363 | thyroid gland | 9,754,177 | 0.97 |  |
| ERR315397 | thyroid gland | 11,026,863 | 0.97 |  |
| ERR315412 | thyroid gland | 18,856,626 | 1.00 |  |
| ERR315422 | thyroid gland | 13,440,117 | 0.96 |  |
| ERR315428 | thyroid gland | 9,719,953 | 0.97 |  |
| ERR315483 | thyroid gland | 7,871,271 | 0.97 |  |
| ERR315491 | thyroid gland | 7,836,954 | 0.97 |  |
| ERR315334 | urinary bladder | 3,896,681 | 1.00 |  |
| ERR315355 | urinary bladder | 12,344,532 | 0.96 |  |
| ERR315370 | urinary bladder | 3,319,098 | 0.95 |  |
| ERR315421 | urinary bladder | 3,123,367 | 0.95 |  |
| ERR315447 | urinary bladder | 11,582,834 | 0.99 |  |
| ERR315453 | urinary bladder | 3,677,518 | 0.99 |  |

**Supplementary Table 3. Genes with high expression divergence between selected cells and tissues, as measured by Phylogenetic ANOVA**

(provided in a separate file).

**Supplementary Table 4. KEGG pathway enrichment of genes with high expression divergence between selected cells and tissues, as measured by Phylogenetic ANOVA**

(provided in a separate file).

**Supplementary Table 5. Genes correlating with cellular turnover in 21 cells and tissues.**

Phylogenetic generalized least squares (PGLS) regression was performed for each cell and tissue against turnover. The Benjamini–Hochberg (BH) false discovery rate procedure (BH-FDR) was used to control the false discovery rate at a level of 5%. Genes highlighted in bold are associated with the cell cycle.

| **Gene** | **modelname** | **modelcoeff** | **p.value** | **BH-FDR** | **description** |
| --- | --- | --- | --- | --- | --- |
| *ABI1* | NULLmodel | -0.084444089 | 0.000362727 | 0.036281805 | abl-interactor 1 |
| *ABRACL* | OU | -0.20767781 | 0.000358 | 0.036281805 | ABRA C-terminal like |
| *ADAT2* | OU | -0.149971601 | 0.000351564 | 0.036281805 | adenosine deaminase, tRNA-specific 2 |
| *ADCY1* | BM | 0.415850334 | 2.77792E-05 | 0.01593204 | adenylate cyclase 1 (brain) |
| *AGA* | BM | -0.132436948 | 0.000482463 | 0.041505613 | aspartylglucosaminidase |
| *AKAP6* | BM | 0.421029278 | 7.47138E-05 | 0.01999673 | A kinase (PRKA) anchor protein 6 |
| *AKTIP* | OU | 0.099623436 | 0.000564324 | 0.042657762 | AKT interacting protein |
| *ALPK1* | OU | -0.158211675 | 0.000265163 | 0.03143022 | alpha-kinase 1 |
| *AMIGO1* | OU | 0.21736438 | 0.000141396 | 0.026789207 | adhesion molecule with Ig-like domain 1 |
| *ANKRD13A* | OU | -0.112812783 | 0.000491201 | 0.041957599 | ankyrin repeat domain 13A |
| *ANKRD46* | OU | 0.164535388 | 0.000156204 | 0.026789207 | ankyrin repeat domain 46 |
| *ANKRD49* | NULLmodel | -0.100090813 | 0.000521685 | 0.042657762 | ankyrin repeat domain 49 |
| *APOBEC3D* | OU | -0.222042238 | 0.000563705 | 0.042657762 | apolipoprotein B mRNA editing enzyme, catalytic polypeptide-like 3D |
| *APOBEC3G* | BM | -0.226927963 | 0.000823354 | 0.048849633 | apolipoprotein B mRNA editing enzyme, catalytic polypeptide-like 3G |
| ***ARF6*** | **BM** | **-0.170305022** | **0.000318299** | **0.034648635** | **ADP-ribosylation factor 6** |
| *ARHGAP11A* | OU | -0.237076578 | 0.000542628 | 0.042657762 | Rho GTPase activating protein 11A |
| ***ARPC4*** | **OU** | **-0.118308498** | **5.92306E-05** | **0.01988454** | **actin related protein 2/3 complex, subunit 4, 20kDa** |
| ***ARPC5*** | **OU** | **-0.109701526** | **0.000805503** | **0.048849633** | **actin related protein 2/3 complex, subunit 5, 16kDa** |
| *B4GAT1* | OU | 0.212804468 | 3.97616E-05 | 0.019155547 | beta-1,4-glucuronyltransferase 1 |
| *BAX* | OU | -0.161094472 | 3.48142E-05 | 0.018230557 | BCL2-associated X protein |
| *BAZ1A* | OU | -0.157572723 | 0.000164604 | 0.026907312 | bromodomain adjacent to zinc finger domain, 1A |
| *BCL10* | OU | -0.171212968 | 7.38153E-06 | 0.01308061 | B-cell CLL/lymphoma 10 |
| ***BORA*** | **OU** | **-0.167009741** | **6.66778E-05** | **0.01988454** | **bora, aurora kinase A activator** |
| ***BRCA2*** | **NULLmodel** | **-0.303723419** | **7.03573E-05** | **0.01988454** | **breast cancer 2, early onset** |
| *C1orf112* | NULLmodel | -0.099067122 | 0.000392089 | 0.037157285 | chromosome 1 open reading frame 112 |
| *C6orf106* | OU | 0.083735466 | 0.000831648 | 0.048952039 | chromosome 6 open reading frame 106 |
| *CAND2* | OU | 0.27155637 | 0.000833209 | 0.048952039 | cullin-associated and neddylation-dissociated 2 (putative) |
| *CAPZA1* | NULLmodel | -0.129979765 | 0.000236114 | 0.029459149 | capping protein (actin filament) muscle Z-line, alpha 1 |
| *CASP1* | NULLmodel | -0.269922495 | 0.000236952 | 0.029459149 | caspase 1, apoptosis-related cysteine peptidase |
| *CASP4* | OU | -0.245781655 | 0.000720029 | 0.047911753 | caspase 4, apoptosis-related cysteine peptidase |
| *CASP8* | BM | -0.228947162 | 0.000171478 | 0.02717481 | caspase 8, apoptosis-related cysteine peptidase |
| *CCDC109B* | OU | -0.236798629 | 0.000765059 | 0.047935988 | coiled-coil domain containing 109B |
| *CCDC91* | OU | 0.089586241 | 0.000197104 | 0.02785271 | coiled-coil domain containing 91 |
| *CCDC92* | NULLmodel | 0.132336979 | 8.91611E-06 | 0.01308061 | coiled-coil domain containing 92 |
| ***CCNA2*** | **OU** | **-0.279574402** | **0.000690852** | **0.046745079** | **cyclin A2** |
| ***CCNB2*** | **NULLmodel** | **-0.414734956** | **1.6123E-05** | **0.01308061** | **cyclin B2** |
| ***CDC20*** | **OU** | **-0.341329322** | **0.000549845** | **0.042657762** | **cell division cycle 20** |
| ***CDC42*** | **NULLmodel** | **-0.08744321** | **0.000346871** | **0.036281805** | **cell division cycle 42** |
| ***CDCA3*** | **OU** | **-0.297274982** | **3.63932E-05** | **0.018263319** | **cell division cycle associated 3** |
| ***CDCA8*** | **OU** | **-0.341278686** | **3.2371E-05** | **0.017721634** | **cell division cycle associated 8** |
| *CDIP1* | OU | 0.12517282 | 0.00055196 | 0.042657762 | cell death-inducing p53 target 1 |
| ***CENPE*** | **NULLmodel** | **-0.266675866** | **0.000744551** | **0.047935988** | **centromere protein E, 312kDa** |
| ***CENPM*** | **NULLmodel** | **-0.291939257** | **0.000150601** | **0.026789207** | **centromere protein M** |
| ***CENPU*** | **NULLmodel** | **-0.296766113** | **0.000453375** | **0.040296078** | **centromere protein U** |
| ***CENPW*** | **OU** | **-0.323969352** | **1.84632E-05** | **0.01308061** | **centromere protein W** |
| ***CHEK1*** | **OU** | **-0.291762984** | **0.000349651** | **0.036281805** | **checkpoint kinase 1** |
| ***CHEK2*** | **NULLmodel** | **-0.249802785** | **2.70752E-05** | **0.01593204** | **checkpoint kinase 2** |
| ***CLIC1*** | **NULLmodel** | **-0.255729901** | **0.000205827** | **0.028170258** | **chloride intracellular channel 1** |
| ***CLSPN*** | **NULLmodel** | **-0.294708469** | **0.000711187** | **0.047586311** | **claspin** |
| *CNOT10* | OU | -0.057003397 | 0.000797086 | 0.048849633 | CCR4-NOT transcription complex, subunit 10 |
| *CPE* | BM | 0.425600798 | 0.000663075 | 0.046175622 | carboxypeptidase E |
| *CRELD1* | NULLmodel | 0.128167764 | 0.000134406 | 0.026789207 | cysteine-rich with EGF-like domains 1 |
| *CRY2* | OU | 0.133396362 | 0.000420076 | 0.038718001 | cryptochrome circadian clock 2 |
| *CYP2R1* | OU | -0.083986149 | 0.00023642 | 0.029459149 | cytochrome P450, family 2, subfamily R, polypeptide 1 |
| *DDB2* | NULLmodel | -0.158125809 | 1.08399E-05 | 0.01308061 | damage-specific DNA binding protein 2, 48kDa |
| *DDX43* | OU | -0.220738417 | 0.000460966 | 0.040459392 | DEAD (Asp-Glu-Ala-Asp) box polypeptide 43 |
| *DDX60L* | OU | -0.200404217 | 0.000144512 | 0.026789207 | DEAD (Asp-Glu-Ala-Asp) box polypeptide 60-like |
| *DPP3* | OU | -0.100116891 | 0.00082272 | 0.048849633 | dipeptidyl-peptidase 3 |
| *EFNA4* | OU | -0.228801235 | 0.00045502 | 0.040296078 | ephrin-A4 |
| *EIF3D* | OU | -0.097790493 | 0.000674281 | 0.046175622 | eukaryotic translation initiation factor 3, subunit D |
| *EIF4A1* | OU | -0.155083478 | 0.00053464 | 0.042657762 | eukaryotic translation initiation factor 4A1 |
| *ELF4* | BM | -0.332603026 | 0.000109502 | 0.02536244 | E74-like factor 4 (ets domain transcription factor) |
| *EPSTI1* | OU | -0.233881813 | 0.000161315 | 0.026907312 | epithelial stromal interaction 1 (breast) |
| ***ESPL1*** | **NULLmodel** | **-0.394938432** | **7.10621E-05** | **0.01988454** | **extra spindle pole bodies homolog 1 (S. cerevisiae)** |
| *ETV7* | OU | -0.255529986 | 0.000252432 | 0.030402917 | ets variant 7 |
| *EXO1* | NULLmodel | -0.360239283 | 6.46286E-05 | 0.01988454 | exonuclease 1 |
| *EZH2* | NULLmodel | -0.237434785 | 0.000113478 | 0.025787268 | enhancer of zeste 2 polycomb repressive complex 2 subunit |
| ***FANCD2*** | **NULLmodel** | **-0.27368503** | **2.61642E-06** | **0.01308061** | **Fanconi anemia, complementation group D2** |
| ***FANCG*** | **NULLmodel** | **-0.124965518** | **6.12434E-05** | **0.01988454** | **Fanconi anemia, complementation group G** |
| *FGD3* | OU | 0.211728507 | 1.17797E-05 | 0.01308061 | FYVE, RhoGEF and PH domain containing 3 |
| ***FOXM1*** | **OU** | **-0.321835475** | **6.04843E-05** | **0.01988454** | **forkhead box M1** |
| *GALNT3* | BM | -0.434447221 | 0.000564879 | 0.042657762 | polypeptide N-acetylgalactosaminyltransferase 3 |
| *GAS8* | OU | 0.126818094 | 6.91672E-05 | 0.01988454 | growth arrest-specific 8 |
| ***GEN1*** | **NULLmodel** | **-0.13831761** | **0.000752656** | **0.047935988** | **GEN1 Holliday junction 5' flap endonuclease** |
| *GPRC5B* | OU | 0.374289605 | 0.000176887 | 0.027289891 | G protein-coupled receptor, class C, group 5, member B |
| *HABP4* | OU | 0.182047949 | 2.6455E-05 | 0.01593204 | hyaluronan binding protein 4 |
| *HEY1* | BM | 0.254152329 | 5.86246E-05 | 0.01988454 | hes-related family bHLH transcription factor with YRPW motif 1 |
| *HNRNPF* | OU | -0.101073096 | 0.000367352 | 0.036281805 | heterogeneous nuclear ribonucleoprotein F |
| *HOMER1* | BM | 0.303606287 | 0.000480767 | 0.041505613 | homer homolog 1 (Drosophila) |
| *HPSE* | NULLmodel | -0.28947244 | 0.000215433 | 0.028829776 | heparanase |
| *IGIP* | NULLmodel | 0.190307275 | 0.000280863 | 0.03143022 | IgA-inducing protein |
| *IKBKE* | OU | -0.285499504 | 4.60432E-05 | 0.019805135 | inhibitor of kappa light polypeptide gene enhancer in B-cells, kinase epsilon |
| *IL10RB* | OU | -0.165098126 | 0.000101887 | 0.024439959 | interleukin 10 receptor, beta |
| ***IQGAP3*** | **OU** | **-0.361142455** | **0.000280306** | **0.03143022** | **IQ motif containing GTPase activating protein 3** |
| *IQSEC3* | BM | 0.38582873 | 0.000463583 | 0.040459392 | IQ motif and Sec7 domain 3 |
| *IRAK4* | OU | -0.165715575 | 0.000201194 | 0.02785271 | interleukin-1 receptor-associated kinase 4 |
| ***JTB*** | **OU** | **-0.096344392** | **7.62906E-10** | **9.18844E-06** | **jumping translocation breakpoint** |
| *KCNAB1* | BM | 0.217595112 | 0.00081679 | 0.048849633 | potassium channel, voltage gated subfamily A regulatory beta subunit 1 |
| *KCNK6* | OU | -0.293737493 | 0.000776468 | 0.047957865 | potassium channel, two pore domain subfamily K, member 6 |
| *KIAA1279* | BM | 0.110177621 | 0.000401147 | 0.037452839 | KIAA1279 |
| ***KIF11*** | **OU** | **-0.274477072** | **0.00022224** | **0.029413791** | **kinesin family member 11** |
| ***KIF23*** | **NULLmodel** | **-0.324426132** | **6.12107E-05** | **0.01988454** | **kinesin family member 23** |
| *KLF3* | OU | -0.147075609 | 0.000268589 | 0.03143022 | Kruppel-like factor 3 (basic) |
| ***KNTC1*** | **OU** | **-0.155764202** | **0.000157001** | **0.026789207** | **kinetochore associated 1** |
| *LBR* | NULLmodel | -0.19539924 | 0.000122467 | 0.026789207 | lamin B receptor |
| *LNP1* | OU | 0.190210809 | 0.000451239 | 0.040296078 | leukemia NUP98 fusion partner 1 |
| *LRP1B* | BM | 0.522581827 | 0.00024215 | 0.029459149 | low density lipoprotein receptor-related protein 1B |
| *LRR1* | OU | -0.167998076 | 0.000355462 | 0.036281805 | leucine rich repeat protein 1 |
| ***MAPRE3*** | **OU** | **0.169395492** | **0.000189525** | **0.027837042** | **microtubule-associated protein, RP/EB family, member 3** |
| ***MASTL*** | **OU** | **-0.107809099** | **0.000566075** | **0.042657762** | **microtubule associated serine/threonine kinase-like** |
| *MB21D1* | BM | -0.199064257 | 0.000604791 | 0.044146077 | Mab-21 domain containing 1 |
| *MCTP2* | OU | -0.349248077 | 0.000370786 | 0.036281805 | multiple C2 domains, transmembrane 2 |
| *MDH1* | BM | 0.170145358 | 0.000157924 | 0.026789207 | malate dehydrogenase 1, NAD (soluble) |
| ***MIS18A*** | **NULLmodel** | **-0.130028404** | **0.000276754** | **0.03143022** | **MIS18 kinetochore protein A** |
| ***MIS18BP1*** | **OU** | **-0.159738154** | **0.000535919** | **0.042657762** | **MIS18 binding protein 1** |
| ***MKI67*** | **OU** | **-0.461708355** | **0.000240354** | **0.029459149** | **marker of proliferation Ki-67** |
| ***MMS22L*** | **NULLmodel** | **-0.174471855** | **0.000123896** | **0.026789207** | **MMS22-like, DNA repair protein** |
| ***MOB1A*** | **OU** | **-0.115651908** | **0.000226082** | **0.029459149** | **MOB kinase activator 1A** |
| *MOV10* | OU | -0.123139675 | 0.000369679 | 0.036281805 | Mov10 RISC complex RNA helicase |
| *MRAS* | OU | 0.233745318 | 0.000165322 | 0.026907312 | muscle RAS oncogene homolog |
| *MSH5* | BM | -0.217575548 | 1.37637E-05 | 0.01308061 | mutS homolog 5 |
| *MTMR8* | OU | -0.132834202 | 0.000177545 | 0.027289891 | myotubularin related protein 8 |
| *NADSYN1* | OU | -0.134811158 | 9.4812E-06 | 0.01308061 | NAD synthetase 1 |
| ***NCAPD2*** | **OU** | **-0.113886173** | **0.000674768** | **0.046175622** | **non-SMC condensin I complex, subunit D2** |
| ***NCAPG*** | **NULLmodel** | **-0.288003487** | **9.21417E-05** | **0.022648044** | **non-SMC condensin I complex, subunit G** |
| ***NCAPH*** | **OU** | **-0.371488117** | **5.99247E-05** | **0.01988454** | **non-SMC condensin I complex, subunit H** |
| *NDUFA4* | BM | 0.140989668 | 0.000594831 | 0.043683821 | NDUFA4, mitochondrial complex associated |
| *NEIL2* | OU | 0.10915862 | 6.85932E-05 | 0.01988454 | nei endonuclease VIII-like 2 (E. coli) |
| *NFKB1* | OU | -0.097285634 | 0.00049732 | 0.042181104 | nuclear factor of kappa light polypeptide gene enhancer in B-cells 1 |
| *NGRN* | BM | 0.121491205 | 0.000539276 | 0.042657762 | neugrin, neurite outgrowth associated |
| *NR2C2AP* | NULLmodel | -0.101272423 | 0.000855457 | 0.049534254 | nuclear receptor 2C2-associated protein |
| *NRAS* | NULLmodel | -0.139148132 | 0.000771886 | 0.047935988 | neuroblastoma RAS viral (v-ras) oncogene homolog |
| ***NUF2*** | **NULLmodel** | **-0.317845595** | **8.76342E-05** | **0.022316629** | **NUF2, NDC80 kinetochore complex component** |
| *NXT2* | OU | -0.145349215 | 0.000701826 | 0.047222317 | nuclear transport factor 2-like export factor 2 |
| ***ORC1*** | **OU** | **-0.343455263** | **2.61248E-05** | **0.01593204** | **origin recognition complex, subunit 1** |
| *PARP16* | BM | -0.090492523 | 0.000743989 | 0.047935988 | poly (ADP-ribose) polymerase family, member 16 |
| ***PARPBP*** | **NULLmodel** | **-0.27258258** | **1.63769E-05** | **0.01308061** | **PARP1 binding protein** |
| *PFN1* | NULLmodel | -0.185594005 | 0.00010349 | 0.024439959 | profilin 1 |
| *PGLS* | OU | -0.125837849 | 0.000755647 | 0.047935988 | 6-phosphogluconolactonase |
| ***PLK4*** | **NULLmodel** | **-0.269347009** | **0.00014897** | **0.026789207** | **polo-like kinase 4** |
| ***PRIM2*** | **NULLmodel** | **-0.127410451** | **0.000668305** | **0.046175622** | **primase, DNA, polypeptide 2 (58kDa)** |
| *PSMB8* | BM | -0.223874342 | 0.000394896 | 0.037157285 | proteasome (prosome, macropain) subunit, beta type, 8 |
| *PTBP3* | OU | -0.159741706 | 0.000270336 | 0.03143022 | polypyrimidine tract binding protein 3 |
| ***PYCARD*** | **OU** | **-0.289115042** | **0.000586753** | **0.043354948** | **PYD and CARD domain containing** |
| *R3HCC1L* | NULLmodel | -0.067272077 | 0.000544393 | 0.042657762 | R3H domain and coiled-coil containing 1-like |
| *RAB32* | OU | -0.280819599 | 0.000151097 | 0.026789207 | RAB32, member RAS oncogene family |
| ***RAB8A*** | **BM** | **-0.159668356** | **0.000752843** | **0.047935988** | **RAB8A, member RAS oncogene family** |
| *RABEP1* | OU | 0.087884772 | 0.000841533 | 0.048963426 | rabaptin, RAB GTPase binding effector protein 1 |
| ***RAD51*** | **OU** | **-0.278731886** | **0.000629113** | **0.044570787** | **RAD51 recombinase** |
| ***RAD51AP1*** | **OU** | **-0.254266064** | **0.000180333** | **0.027289891** | **RAD51 associated protein 1** |
| *RBM3* | NULLmodel | -0.143774153 | 0.000772134 | 0.047935988 | RNA binding motif (RNP1, RRM) protein 3 |
| ***RCC1*** | **OU** | **-0.186578378** | **1.79585E-05** | **0.01308061** | **regulator of chromosome condensation 1** |
| ***RCC2*** | **BM** | **-0.156240425** | **7.26436E-05** | **0.01988454** | **regulator of chromosome condensation 2** |
| ***RFWD3*** | **OU** | **-0.14594044** | **0.00020002** | **0.02785271** | **ring finger and WD repeat domain 3** |
| *RGS10* | BM | -0.18471598 | 0.000139343 | 0.026789207 | regulator of G-protein signaling 10 |
| *RHNO1* | BM | -0.108165027 | 0.00081292 | 0.048849633 | RAD9-HUS1-RAD1 interacting nuclear orphan 1 |
| *RIPK1* | NULLmodel | -0.077936252 | 0.000817279 | 0.048849633 | receptor (TNFRSF)-interacting serine-threonine kinase 1 |
| *RNPEP* | OU | -0.129108658 | 0.000196456 | 0.02785271 | arginyl aminopeptidase (aminopeptidase B) |
| *RP2* | OU | -0.17684134 | 0.000322306 | 0.034659379 | retinitis pigmentosa 2 (X-linked recessive) |
| *RPL22L1* | OU | -0.181383301 | 0.000365982 | 0.036281805 | ribosomal protein L22-like 1 |
| *SAMD9* | OU | -0.266022491 | 0.000181268 | 0.027289891 | sterile alpha motif domain containing 9 |
| *SCN4B* | BM | 0.349896807 | 0.000572933 | 0.042817631 | sodium channel, voltage gated, type IV beta subunit |
| *SECTM1* | NULLmodel | -0.350161313 | 0.000193588 | 0.02785271 | secreted and transmembrane 1 |
| *SENP2* | OU | 0.05937254 | 0.000760494 | 0.047935988 | SUMO1/sentrin/SMT3 specific peptidase 2 |
| ***SEPT4*** | **OU** | **0.349377212** | **0.000444061** | **0.04021254** | **septin 4** |
| *SERPINI1* | OU | 0.227664959 | 0.000737557 | 0.047935988 | serpin peptidase inhibitor, clade I (neuroserpin), member 1 |
| ***SLBP*** | **OU** | **-0.081907985** | **0.000389748** | **0.037157285** | **stem-loop binding protein** |
| *SLC10A7* | NULLmodel | -0.09622261 | 0.000242126 | 0.029459149 | solute carrier family 10, member 7 |
| *SLC1A5* | OU | -0.328606867 | 0.000839102 | 0.048963426 | solute carrier family 1 (neutral amino acid transporter), member 5 |
| *SLC41A1* | OU | 0.193915629 | 0.000652513 | 0.045958303 | solute carrier family 41 (magnesium transporter), member 1 |
| *SLC4A1AP* | OU | 0.044718974 | 0.000185433 | 0.027572286 | solute carrier family 4 (anion exchanger), member 1, adaptor protein |
| *SMPD2* | OU | -0.140493491 | 4.85266E-05 | 0.01988454 | sphingomyelin phosphodiesterase 2, neutral membrane (neutral sphingomyelinase) |
| *SNRPB* | OU | -0.102719387 | 0.000549241 | 0.042657762 | small nuclear ribonucleoprotein polypeptides B and B1 |
| *SNRPN* | OU | 0.245803452 | 1.19193E-05 | 0.01308061 | small nuclear ribonucleoprotein polypeptide N |
| *SNTA1* | BM | 0.254986533 | 0.000761145 | 0.047935988 | syntrophin, alpha 1 |
| *SNURF* | NULLmodel | 0.242376773 | 3.67246E-06 | 0.01308061 | SNRPN upstream reading frame |
| *SP1* | NULLmodel | -0.099288376 | 0.000149524 | 0.026789207 | Sp1 transcription factor |
| *SP140L* | BM | -0.216923247 | 0.000565183 | 0.042657762 | SP140 nuclear body protein-like |
| *SPIN4* | OU | -0.190267248 | 0.000154275 | 0.026789207 | spindlin family, member 4 |
| ***SRC*** | **OU** | **-0.180528337** | **0.000132965** | **0.026789207** | **SRC proto-oncogene, non-receptor tyrosine kinase** |
| ***STIL*** | **OU** | **-0.218206233** | **0.000820475** | **0.048849633** | **SCL/TAL1 interrupting locus** |
| *STK26* | OU | -0.333528599 | 7.97869E-06 | 0.01308061 | serine/threonine protein kinase 26 |
| ***STK38*** | **OU** | **-0.130669927** | **4.25234E-05** | **0.019698134** | **serine/threonine kinase 38** |
| *STXBP1* | BM | 0.26686139 | 0.000624531 | 0.044508012 | syntaxin binding protein 1 |
| *TAP1* | NULLmodel | -0.177951017 | 0.000319329 | 0.034648635 | transporter 1, ATP-binding cassette, sub-family B (MDR/TAP) |
| *TARSL2* | OU | 0.134166054 | 0.000424342 | 0.038718001 | threonyl-tRNA synthetase-like 2 |
| *TBX19* | OU | -0.233500089 | 0.000575926 | 0.042817631 | T-box 19 |
| ***TCF19*** | **OU** | **-0.172047486** | **0.000623748** | **0.044508012** | **transcription factor 19** |
| *TDP1* | NULLmodel | -0.121271182 | 0.000232997 | 0.029459149 | tyrosyl-DNA phosphodiesterase 1 |
| *TEX2* | NULLmodel | 0.101927464 | 0.000388139 | 0.037157285 | testis expressed 2 |
| *THOC6* | BM | -0.140152416 | 0.000373542 | 0.036281805 | THO complex 6 homolog (Drosophila) |
| *TK1* | NULLmodel | -0.267170328 | 0.000743945 | 0.047935988 | thymidine kinase 1, soluble |
| *TMEM178A* | NULLmodel | 0.260468692 | 0.00051169 | 0.042657762 | transmembrane protein 178A |
| *TMEM194A* | OU | -0.112844561 | 0.000354319 | 0.036281805 | transmembrane protein 194A |
| *TMPO* | NULLmodel | -0.132749808 | 5.09899E-05 | 0.01988454 | thymopoietin |
| *TNFRSF10A* | OU | -0.22149214 | 0.000566692 | 0.042657762 | tumor necrosis factor receptor superfamily, member 10a |
| *TOR3A* | OU | -0.101336171 | 0.000281839 | 0.03143022 | torsin family 3, member A |
| *TP53* | OU | -0.19527094 | 4.60283E-05 | 0.019805135 | tumor protein p53 |
| ***TPPP*** | **NULLmodel** | **0.251303698** | **0.000667449** | **0.046175622** | **tubulin polymerization promoting protein** |
| *TRAF7* | OU | -0.081671692 | 0.000421689 | 0.038718001 | TNF receptor-associated factor 7, E3 ubiquitin protein ligase |
| *TRAPPC2* | OU | -0.080684945 | 1.51975E-05 | 0.01308061 | trafficking protein particle complex 2 |
| *TRIM22* | BM | -0.215289087 | 0.000686704 | 0.046726943 | tripartite motif containing 22 |
| *TRIM25* | BM | -0.13296348 | 0.000609407 | 0.044215076 | tripartite motif containing 25 |
| *TRIM38* | BM | -0.201482021 | 8.69388E-05 | 0.022316629 | tripartite motif containing 38 |
| ***TRIP13*** | **NULLmodel** | **-0.25789886** | **0.00031167** | **0.034438145** | **thyroid hormone receptor interactor 13** |
| *TTC7B* | OU | 0.135703684 | 0.0002766 | 0.03143022 | tetratricopeptide repeat domain 7B |
| ***TTF2*** | **OU** | **-0.126653839** | **0.000566176** | **0.042657762** | **transcription termination factor, RNA polymerase II** |
| *UBE2J1* | NULLmodel | -0.147708235 | 1.22337E-05 | 0.01308061 | ubiquitin-conjugating enzyme E2, J1 |
| *UBR3* | BM | 0.117299252 | 0.000208422 | 0.028204897 | ubiquitin protein ligase E3 component n-recognin 3 (putative) |
| *VASP* | OU | -0.227216768 | 8.89404E-05 | 0.022316629 | vasodilator-stimulated phosphoprotein |
| *WDR55* | OU | -0.098020476 | 0.000133552 | 0.026789207 | WD repeat domain 55 |
| *WDR60* | OU | 0.091223421 | 0.000136743 | 0.026789207 | WD repeat domain 60 |
| *ZC3HAV1* | OU | -0.183783762 | 0.000169726 | 0.02717481 | zinc finger CCCH-type, antiviral 1 |
| *ZNF101* | OU | -0.140752627 | 0.000618396 | 0.044508012 | zinc finger protein 101 |
| *ZNF217* | OU | -0.205014601 | 7.05667E-05 | 0.01988454 | zinc finger protein 217 |
| *ZNF385B* | OU | 0.360867925 | 0.000560774 | 0.042657762 | zinc finger protein 385B |
| *ZNF680* | NULLmodel | 0.148534711 | 0.000765004 | 0.047935988 | zinc finger protein 680 |
| ***ZWINT*** | **OU** | **-0.271706856** | **0.000155113** | **0.026789207** | **ZW10 interacting kinetochore protein** |

**Supplementary Table 6. KEGG pathway enrichment of genes correlating with cellular turnover in 21 cells and tissues.**

The bootstrap value is defined as the number of times (out of 5,000) that *P*-values of a random gene list were smaller than the *P*-value of each gene set.

| **Pathway Name** | **Count** | **Pathway**  **Size** | **Adj *P*-value** | **Bootstrap**  ***P*-value** | **genes** |
| --- | --- | --- | --- | --- | --- |
| KEGG_P53_SIGNALING_PATHWAY | 7 | 67 | 2.5E-03 | 0.0E+00 | *CASP8,BAX,CHEK2,DDB2,CHEK1,TP53,CCNB2* |
| KEGG_APOPTOSIS | 7 | 86 | 6.6E-03 | 0.0E+00 | *CASP8,RIPK1,IRAK4,BAX,NFKB1,TNFRSF10A,TP53* |
| KEGG_CELL_CYCLE | 8 | 124 | 6.8E-03 | 2.0E-04 | *CCNA2,CHEK1,CDC20,ESPL1,CHEK2,TP53,ORC1,CCNB2* |
| KEGG_CYTOSOLIC_DNA_SENSING_PATHWAY | 5 | 56 | 2.5E-02 | 0.0E+00 | *RIPK1,CASP1,IKBKE,NFKB1,PYCARD* |
| KEGG_RIG_I_LIKE_RECEPTOR_SIGNALING_PATHWAY | 5 | 70 | 4.6E-02 | 1.0E-03 | *CASP8,TRIM25,RIPK1,IKBKE,NFKB1* |
| KEGG_PANCREATIC_CANCER | 5 | 70 | 4.6E-02 | 6.0E-04 | *RAD51,BRCA2,NFKB1,TP53,CDC42* |

**Supplementary Table 7. Biological process (BP) Gene Ontology (GO) term enrichment of genes correlating with cellular turnover in 21 cells and tissues.**

The bootstrap value is defined as the number of times (out of 5,000) that *P*-values of a random gene list were smaller than the *P*-value of each gene set.

| **Gene Ontology**  **(GO) term** | **Count** | **GO term**  **size** | **Adj Pvalue** | **Bootstrap**  **P Value** | **Genes** |
| --- | --- | --- | --- | --- | --- |
| M_PHASE | 11 | 114 | 5.9E-04 | 0.0E+00 | *KNTC1,CCNA2,ESPL1,ZWINT,CHEK1,RCC1,KIF11,RAD51,NCAPH,MSH5,CENPE* |
| CELL_CYCLE_GO_0007049 | 17 | 313 | 9.6E-04 | 0.0E+00 | *CCNA2,ESPL1,ZWINT,CHEK1,CHEK2,RCC1,CDC20,FANCG,TP53,RAD51,NCAPH,KNTC1,BRCA2,KIF23,KIF11,MSH5,CENPE* |
| CELL_CYCLE_PROCESS | 13 | 193 | 1.1E-03 | 0.0E+00 | *CCNA2,ESPL1,ZWINT,CHEK1,RCC1,RAD51,NCAPH,KNTC1,BRCA2,KIF23,KIF11,MSH5,CENPE* |
| MITOSIS | 8 | 82 | 4.8E-03 | 0.0E+00 | *KNTC1,CCNA2,KIF11,ESPL1,ZWINT,RCC1,NCAPH,CENPE* |
| M_PHASE_OF_MITOTIC_CELL_CYCLE | 8 | 85 | 4.8E-03 | 0.0E+00 | *KNTC1,CCNA2,KIF11,ESPL1,ZWINT,RCC1,NCAPH,CENPE* |
| CELL_CYCLE_PHASE | 11 | 170 | 4.8E-03 | 0.0E+00 | *KNTC1,CCNA2,ESPL1,ZWINT,CHEK1,RCC1,KIF11,RAD51,NCAPH,MSH5,CENPE* |
| CELL_CYCLE_CHECKPOINT_GO_0000075 | 6 | 48 | 7.2E-03 | 0.0E+00 | *KNTC1,CHEK1,CHEK2,FANCG,CCNA2,ZWINT* |
| MITOTIC_SISTER_CHROMATID_SEGREGATION | 4 | 16 | 7.2E-03 | 0.0E+00 | *ESPL1,CENPE,NCAPH,ZWINT* |
| SISTER_CHROMATID_SEGREGATION | 4 | 17 | 8.3E-03 | 0.0E+00 | *CENPE,ESPL1,NCAPH,ZWINT* |
| RESPONSE_TO_DNA_DAMAGE_STIMULUS | 10 | 159 | 8.5E-03 | 0.0E+00 | *CCNA2,CHEK1,CHEK2,BRCA2,FANCG,TP53,RAD51,DDB2,EXO1,MSH5* |
| POSITIVE_REGULATION_OF_I_KAPPAB_KINASE_NF_KAPPAB_CASCADE | 7 | 87 | 2.0E-02 | 4.0E-04 | *CASP8,CASP1,BCL10,TRIM38,RIPK1,SECTM1,IKBKE* |
| MITOTIC_SPINDLE_ORGANIZATION_AND_BIOGENESIS | 3 | 10 | 2.2E-02 | 2.0E-04 | *KIF23,KIF11,RCC1* |
| MITOTIC_CELL_CYCLE | 9 | 153 | 2.2E-02 | 2.0E-04 | *KNTC1,CCNA2,ESPL1,ZWINT,RCC1,KIF23,KIF11,NCAPH,CENPE* |
| REGULATION_OF_I_KAPPAB_KINASE_NF_KAPPAB_CASCADE | 7 | 93 | 2.2E-02 | 1.0E-03 | *CASP8,CASP1,BCL10,TRIM38,RIPK1,SECTM1,IKBKE* |
| ORGANELLE_ORGANIZATION_AND_BIOGENESIS | 17 | 471 | 2.2E-02 | 2.0E-04 | *ESPL1,ZWINT,ARF6,RCC1,EZH2,TP53,MRAS,ARPC4,ARPC5,KIF23,CDC42,KIF11,BAX,NCAPH,BRCA2,TPPP,CENPE* |
| NUCLEOBASENUCLEOSIDENUCLEOTIDE_AND_NUCLEIC_ACID_METABOLIC_PROCESS | 32 | 1230 | 2.2E-02 | 2.0E-04 | *HNRNPF,ELF4,EZH2,TP53,TTF2,TRIM22,BCL10,DDB2,EXO1,SLBP,BAX,TK1,TCF19,FANCG,RAD51,ETV7,APOBEC3G,BRCA2,PYCARD,ORC1,TRAPPC2,TRIP13,RBM3,PRIM2,PGLS,CHEK1,NFKB1,TBX19,SNRPN,SNRPB,MSH5,SP1* |
| POSITIVE_REGULATION_OF_SIGNAL_TRANSDUCTION | 8 | 125 | 2.2E-02 | 4.0E-04 | *BCL10,TRIM38,TRAF7,IKBKE,CASP8,CASP1,RIPK1,SECTM1* |
| SPINDLE_ORGANIZATION_AND_BIOGENESIS | 3 | 11 | 2.2E-02 | 4.0E-04 | *KIF23,KIF11,RCC1* |
| POSITIVE_REGULATION_OF_CELLULAR_PROCESS | 21 | 666 | 2.3E-02 | 6.0E-04 | *ARF6,ELF4,TP53,RIPK1,BCL10,TRAF7,IKBKE,CDC42,CASP4,CASP8,CASP1,SECTM1,BAX,CHEK2,RAD51,AMIGO1,TRIM38,AKTIP,TPPP,PYCARD,SP1* |
| RESPONSE_TO_ENDOGENOUS_STIMULUS | 10 | 196 | 2.3E-02 | 0.0E+00 | *CCNA2,CHEK1,CHEK2,FANCG,TP53,RAD51,BRCA2,DDB2,EXO1,MSH5* |
| POSITIVE_REGULATION_OF_BIOLOGICAL_PROCESS | 21 | 706 | 4.2E-02 | 6.0E-04 | *ARF6,ELF4,TP53,RIPK1,BCL10,TRAF7,IKBKE,CDC42,CASP4,CASP8,CASP1,SECTM1,BAX,CHEK2,RAD51,AMIGO1,TRIM38,AKTIP,TPPP,PYCARD,SP1* |
| CHROMOSOME_SEGREGATION | 4 | 32 | 4.2E-02 | 0.0E+00 | *NCAPH,CENPE,ESPL1,ZWINT* |
| DNA_METABOLIC_PROCESS | 11 | 255 | 4.2E-02 | 1.0E-03 | *PRIM2,ORC1,CHEK1,FANCG,TP53,RAD51,BRCA2,DDB2,EXO1,MSH5,BAX* |
| DNA_DEPENDENT_DNA_REPLICATION | 5 | 55 | 4.2E-02 | 8.0E-04 | *PRIM2,ORC1,RAD51,EXO1,MSH5* |
| I_KAPPAB_KINASE_NF_KAPPAB_CASCADE | 7 | 114 | 4.5E-02 | 1.8E-03 | *BCL10,TRIM38,IKBKE,CASP8,CASP1,RIPK1,SECTM1* |
| DNA_DAMAGE_RESPONSESIGNAL_TRANSDUCTION | 4 | 34 | 4.6E-02 | 2.0E-04 | *CHEK1,CHEK2,CCNA2,TP53* |
| BIOPOLYMER_METABOLIC_PROCESS | 38 | 1668 | 4.6E-02 | 1.2E-03 | *EZH2,TRIM22,BCL10,DDB2,SLBP,BAX,UBR3,RAD51,AGA,APOBEC3G,BRCA2,TRAPPC2,TRAF7,PLK4,PRIM2,NFKB1,TBX19,SNRPN,SNRPB,AKTIP,SP1,HNRNPF,ELF4,TP53,TTF2,ABI1,IKBKE,EXO1,TCF19,FANCG,ETV7,ORC1,TRIP13,STK38,RBM3,CHEK1,CDC20,MSH5* |
| MICROTUBULE_CYTOSKELETON_ORGANIZATION_AND_BIOGENESIS | 4 | 35 | 4.8E-02 | 8.0E-04 | *KIF23,RCC1,KIF11,TPPP* |
| MEIOTIC_RECOMBINATION | 3 | 17 | 5.0E-02 | 8.0E-04 | *MSH5,CHEK1,RAD51* |
| POSITIVE_REGULATION_OF_CELLULAR_COMPONENT_ORGANIZATION_AND_BIOGENESIS | 4 | 36 | 5.0E-02 | 1.0E-03 | *ARF6,CDC42,AMIGO1,TPPP* |

**Supplementary Table 8. Genes correlating with cellular turnover in 18 cells and tissues after exclusion of cells and tissues of the immune system (monocyte, bone marrow and spleen).**

Phylogenetic generalized least squares (PGLS) regression was performed for each cell and tissue against turnover. The Benjamini–Hochberg (BH) false discovery rate procedure (BH-FDR) was used to control the false discovery rate at a level of 5%. Genes highlighted in bold are associated with the cell cycle.

| **Gene** | **modelname** | **modelcoeff** | **p.value** | **BH-FDR** | **description** |
| --- | --- | --- | --- | --- | --- |
| *ANKRD34A* | OU | 0.1436609743 | 0.0000000002 | 0.0000012025 | ankyrin repeat domain 13A |
| *ANO10* | OU | -0.1245051917 | 0.0002024839 | 0.0452111383 | anoctamin 10 |
| *ARFIP1* | OU | -0.1202123289 | 0.0000538311 | 0.0281887811 | ADP ribosylation factor interacting protein 1 |
| *BCL10* | OU | -0.1932997766 | 0.0000076354 | 0.0114951152 | B-cell CLL/lymphoma 10 |
| *BCL6B* | OU | 0.2550114768 | 0.0000000140 | 0.0000421435 | B-cell CLL/lymphoma 6, member B |
| *BRCA2* | OU | -0.2980048563 | 0.0002361980 | 0.0474128035 | **breast cancer 2, early onset** |
| *BUB1B* | OU | -0.3390160803 | 0.0002038314 | 0.0452111383 | **BUB1B, mitotic checkpoint serine/threonine kinase** |
| *CARD8* | OU | 0.056424165 | 0.0000002339 | 0.0005634390 | caspase recruitment domain family member 8 |
| *CCDC92* | NULLmodel | 0.1333446232 | 0.0000354149 | 0.0213268377 | coiled-coil domain containing 92 |
| *CCNB2* | OU | -0.435498817 | 0.0000208146 | 0.0192839721 | **cyclin B2** |
| *CDC42* | OU | -0.1014790205 | 0.0001769071 | 0.0444211596 | **cell division cycle 42** |
| *CDCA3* | OU | -0.2877559306 | 0.0002350960 | 0.0474128035 | **cell division cycle associated 3** |
| *CDCA8* | OU | -0.3272006589 | 0.0002177222 | 0.0452111383 | **cell division cycle associated 8** |
| *CELF2* | OU | 0.2432088754 | 0.0000109945 | 0.0132417271 | CUGBP, Elav-like family member 2 |
| *CENPF* | OU | -0.3801881376 | 0.0000190385 | 0.0191082860 | **centromere protein E, 312kDa** |
| *CENPW* | OU | -0.3164810408 | 0.0001187897 | 0.0389441710 | **centromere protein W** |
| *CHEK1* | OU | -0.3325429498 | 0.0001107823 | 0.0389441710 | **checkpoint kinase 1** |
| *CHEK2* | NULLmodel | -0.2568122284 | 0.0002413530 | 0.0476533639 | **checkpoint kinase 2** |
| *CNOT1* | OU | -0.0929941235 | 0.0002708546 | 0.0494268693 | CCR4-NOT transcription complex, subunit 10 |
| *CRELD1* | BM | 0.1666556175 | 0.0000649059 | 0.0296092462 | cysteine-rich with EGF-like domains 1 |
| *CRY2* | OU | 0.1626428429 | 0.0001716906 | 0.0444211596 | cryptochrome circadian clock 2 |
| *DDB2* | OU | -0.1765248968 | 0.0000345630 | 0.0213268377 | damage-specific DNA binding protein 2, 48kDa |
| *DDX52* | BM | -0.0833255183 | 0.0000099062 | 0.0132417271 | DEAD (Asp-Glu-Ala-Asp) box polypeptide 52 |
| *DIAPH3* | OU | -0.4014538872 | 0.0001196392 | 0.0389441710 | diaphanous related formin 3 |
| *ECT2* | OU | -0.195576973 | 0.0001291345 | 0.0393195578 | **epithelial cell transforming 2** |
| *EIF5B* | OU | 0.0902823404 | 0.0000026600 | 0.0045767673 | eukaryotic translation initiation factor 5B |
| *EIF6* | NULLmodel | -0.1328672748 | 0.0002126540 | 0.0452111383 | eukaryotic translation initiation factor 6 |
| *EXO1* | OU | -0.3755807361 | 0.0000688358 | 0.0296092462 | exonuclease 1 |
| *FANCD2* | OU | -0.2472341635 | 0.0000305404 | 0.0213268377 | **Fanconi anemia, complementation group D2** |
| *FOXM1* | OU | -0.3375308659 | 0.0001560614 | 0.0417689681 | **forkhead box M1** |
| *GDI2* | OU | -0.1103000261 | 0.0001807237 | 0.0444211596 | GDP dissociation inhibitor 2 |
| *HEY1* | BM | 0.2742827218 | 0.0001360220 | 0.0393195578 | hes-related family bHLH transcription factor with YRPW motif 1 |
| *HNRNPF* | OU | -0.1197013802 | 0.0002589278 | 0.0481845704 | heterogeneous nuclear ribonucleoprotein F |
| *KIF11* | OU | -0.3011893163 | 0.0000678816 | 0.0296092462 | **kinesin family member 11** |
| *KIF23* | NULLmodel | -0.3379788808 | 0.0001403804 | 0.0393195578 | **kinesin family member 23** |
| *MKI67* | OU | -0.5311818156 | 0.0000163897 | 0.0179452371 | **marker of proliferation Ki-67** |
| *MSH5* | NULLmodel | -0.1765892172 | 0.0000263047 | 0.0213268377 | mutS homolog 5 |
| *NADSYN1* | OU | -0.1159951885 | 0.0002157657 | 0.0452111383 | NAD synthetase 1 |
| *NCAPG* | OU | -0.2950453311 | 0.0000801874 | 0.0321925538 | **non-SMC condensin I complex, subunit G** |
| *NCAPH* | OU | -0.3804468431 | 0.0001385565 | 0.0393195578 | **non-SMC condensin I complex, subunit H** |
| *NET1* | OU | -0.1836302141 | 0.0002555646 | 0.0481845704 | neuroepithelial cell transforming 1 |
| *NUF2* | OU | -0.3119194183 | 0.0001056182 | 0.0385474350 | **NUF2, NDC80 kinetochore complex component** |
| *ORC1* | OU | -0.3167835095 | 0.0000939133 | 0.0364868370 | **origin recognition complex, subunit 1** |
| *PARPBP* | NULLmodel | -0.2823140524 | 0.0000307018 | 0.0213268377 | **PARP1 binding protein** |
| *PCDH9* | BM | 0.4116371778 | 0.0002134395 | 0.0452111383 | protocadherin 9 |
| *PDCD6IP* | OU | -0.1025430765 | 0.0000604451 | 0.0291200420 | programmed cell death 6 interacting protein |
| *PLEKHG1* | OU | 0.1728193785 | 0.0000000055 | 0.0000220826 | pleckstrin homology and RhoGEF domain containing G |
| *PLK4* | OU | -0.2621647122 | 0.0001940098 | 0.0452111383 | **polo-like kinase 4** |
| *RARS* | BM | -0.0853635859 | 0.0002600463 | 0.0481845704 | arginyl-tRNA synthetase |
| *RCC1* | OU | -0.2052752502 | 0.0000574400 | 0.0288253162 | **regulator of chromosome condensation 1** |
| *RCCD1* | OU | -0.0769654366 | 0.0000000000 | 0.0000000000 | RCC1 domain containing 1 |
| *SAMD9* | OU | -0.3182530888 | 0.0000444433 | 0.0243306714 | sterile alpha motif domain containing 9 |
| *SCYL2* | NULLmodel | -0.0815295539 | 0.0001778749 | 0.0444211596 | SCY1-like, kinase-like 2 |
| *SERPINE3* | OU | -0.2253616941 | 0.0000006941 | 0.0013933687 | serpin peptidase inhibitor, clade E (nexin, plasminogen activator inhibitor type 1), member 3 |
| *SHANK3* | NULLmodel | 0.2958313235 | 0.0001195130 | 0.0389441710 | SH3 and multiple ankyrin repeat domains 3 |
| *SLC25A43* | OU | -0.2216121269 | 0.0001384529 | 0.0393195578 | **solute carrier family 25 member 43** |
| *SNRPN* | NULLmodel | 0.2399422972 | 0.0001508477 | 0.0412911357 | small nuclear ribonucleoprotein polypeptide N |
| *SPATA18* | BM | -0.3906375489 | 0.0000391750 | 0.0224677681 | spermatogenesis associated 18 |
| *STK26* | BM | -0.315018743 | 0.0001009801 | 0.0380063735 | serine/threonine protein kinase 26 |
| ***STK38*** | **OU** | **-0.1412317673** | **0.0001948938** | **0.0452111383** | **serine/threonine kinase 38** |
| *STOML1* | OU | 0.1414341418 | 0.0000323682 | 0.0213268377 | stomatin like 1 |
| *TP53* | OU | -0.2073183257 | 0.0002488407 | 0.0481845704 | tumor protein p53 |
| *TP53I3* | OU | -0.2303929158 | 0.0000335883 | 0.0213268377 | tumor protein p53 inducible protein 3 |
| *TTYH2* | BM | 0.2736972793 | 0.0000791939 | 0.0321925538 | tweety family member 2 |
| *ZBTB46* | NULLmodel | 0.2585655469 | 0.0001283044 | 0.0393195578 | zinc finger and BTB domain containing 46 |
| *ZWINT* | OU | -0.2887779156 | 0.0002067490 | 0.0452111383 | **ZW10 interacting kinetochore protein** |

**Supplementary Table 9. KEGG pathway enrichment of genes correlating with cellular turnover in 18 cells and tissues after exclusion of cells and tissues of the immune system (monocyte, bone marrow and spleen).**

The bootstrap value is defined as the number of times (out of 5,000) that *P*-values of a random gene list were smaller than the *P*-value of each gene set.

| **Pathway Name** | **Count** | **Pathway**  **Size** | **Adj P-value** | **Bootstrap**  **P-value** | **genes** |
| --- | --- | --- | --- | --- | --- |
| KEGG_P53_SIGNALING_PATHWAY | 6 | 67 | 8.84E-08 | 0 | *TP53I3,CHEK2,DDB2,CHEK1,TP53,CCNB2* |
| KEGG_CELL_CYCLE | 6 | 124 | 3.49E-06 | 0 | *CHEK1,BUB1B,CHEK2,TP53,ORC1,CCNB2* |
| KEGG_PANCREATIC_CANCER | 3 | 70 | 0.001958 | 8.0E-04 | *BRCA2,TP53,CDC42* |
| KEGG_CIRCADIAN_RHYTHM_MAMMAL | 3 | 13 | 0.046674 | 2.9E-02 | *CRY2* |

**Supplementary Table 10. Biological process (BP) Gene Ontology (GO) term enrichment of genes correlating with cellular turnover in 18 cells and tissues after exclusion of cells and tissues of the immune system (monocyte, bone marrow and spleen).**

**The bootstrap value is defined as the number of times (out of 5,000) that *P*-values of a random gene list were smaller than the *P*-value of each gene set.**

| **Gene Ontology**  **(GO) term** | **Count** | **GO term**  **size** | **Adj Pvalue** | **Bootstrap**  **P Value** | **Genes** |
| --- | --- | --- | --- | --- | --- |
| CELL_CYCLE_GO_0007049 | 12 | 313 | 5.6E-06 | 0.0E+00 | *ZWINT,CHEK1,CHEK2,RCC1,TP53,NCAPH,BRCA2,KIF23,KIF11,BUB1B,MSH5,CENPF* |
| CELL_CYCLE_PROCESS | 10 | 193 | 5.6E-06 | 0.0E+00 | *ZWINT,CHEK1,RCC1,NCAPH,BRCA2,KIF23,KIF11,BUB1B,MSH5,CENPF* |
| M_PHASE | 7 | 114 | 2.2E-04 | 0.0E+00 | *ZWINT,CHEK1,RCC1,KIF11,BUB1B,NCAPH,MSH5* |
| CELL_CYCLE_PHASE | 8 | 170 | 2.2E-04 | 0.0E+00 | *ZWINT,CHEK1,RCC1,KIF11,BUB1B,NCAPH,MSH5,CENPF* |
| MITOTIC_CELL_CYCLE | 7 | 153 | 1.1E-03 | 0.0E+00 | *ZWINT,RCC1,KIF23,KIF11,BUB1B,NCAPH,CENPF* |
| RESPONSE_TO_DNA_DAMAGE_STIMULUS | 7 | 159 | 1.2E-03 | 0.0E+00 | *CHEK1,CHEK2,BRCA2,TP53,DDB2,EXO1,MSH5* |
| MITOTIC_SPINDLE_ORGANIZATION_AND_BIOGENESIS | 3 | 10 | 1.5E-03 | 0.0E+00 | *KIF23,KIF11,RCC1* |
| SPINDLE_ORGANIZATION_AND_BIOGENESIS | 3 | 11 | 1.8E-03 | 0.0E+00 | *KIF23,KIF11,RCC1* |
| RESPONSE_TO_ENDOGENOUS_STIMULUS | 7 | 196 | 3.1E-03 | 0.0E+00 | *CHEK1,CHEK2,TP53,BRCA2,DDB2,EXO1,MSH5* |
| MITOSIS | 5 | 82 | 3.5E-03 | 0.0E+00 | *KIF11,ZWINT,BUB1B,RCC1,NCAPH* |
| M_PHASE_OF_MITOTIC_CELL_CYCLE | 5 | 85 | 3.8E-03 | 0.0E+00 | *KIF11,ZWINT,BUB1B,RCC1,NCAPH* |
| CELL_CYCLE_CHECKPOINT_GO_0000075 | 4 | 48 | 5.5E-03 | 0.0E+00 | *CHEK1,CHEK2,ZWINT,BUB1B* |
| NUCLEOTIDE_EXCISION_REPAIR | 3 | 20 | 7.3E-03 | 2.0E-04 | *TP53,BRCA2,DDB2* |
| DNA_METABOLIC_PROCESS | 7 | 255 | 1.1E-02 | 0.0E+00 | *ORC1,CHEK1,TP53,BRCA2,DDB2,EXO1,MSH5* |
| REGULATION_OF_CELL_CYCLE | 6 | 180 | 1.1E-02 | 2.0E-04 | *ZWINT,CHEK1,CHEK2,RCC1,TP53,BUB1B* |
| DNA_REPAIR | 5 | 123 | 1.5E-02 | 0.0E+00 | *BRCA2,TP53,DDB2,EXO1,MSH5* |
| CHROMOSOME_SEGREGATION | 3 | 32 | 2.3E-02 | 4.0E-04 | *NCAPH,CENPF,ZWINT* |
| DNA_DAMAGE_RESPONSESIGNAL_TRANSDUCTION | 3 | 34 | 2.6E-02 | 2.0E-04 | *CHEK1,CHEK2,TP53* |
| MICROTUBULE_CYTOSKELETON_ORGANIZATION_AND_BIOGENESIS | 3 | 35 | 2.7E-02 | 2.0E-04 | *KIF23,RCC1,KIF11* |
| REGULATION_OF_MITOSIS | 3 | 41 | 4.1E-02 | 8.0E-04 | *RCC1,ZWINT,BUB1B* |

**Supplementary Table 11. LINCS analysis of genes correlating with cellular turnover in 21 cells and tissues.**

**The metric score_best2/4/6 denotes the mean connectivity score across the 2/4/6 cell lines in which the perturbagen (overexpression or shRNA knockdown) connected most strongly to the query; ncell number of cell lines over which the connectivity between the query and the perturbagen have been summarized; nsig the total number of perturbagen signatures over which its connectivity to the query has been summarized. Scores greater than 90 in the score_best4 metric are considered significant connections.**

| **cmap_id** | **cmap_name** | **score_best2** | **score_best4** | **score_best6** | **ncell** | **nsig** | **category** |
| --- | --- | --- | --- | --- | --- | --- | --- |
| ccsbBroad304_05980 | *CDKN1B* | 100 | 99.529 | 97.425 | 8 | 8 | Overexpression_connections |
| ccsbBroad304_00282 | *CDKN1A* | 97.651 | 96.849 | 94.128 | 7 | 7 | Overexpression_connections |
| ccsbBroad304_00283 | *CDKN2C* | 98.48 | 96.722 | 94.542 | 7 | 7 | Overexpression_connections |
| ccsbBroad304_06021 | *KLF6* | 97.913 | 96.206 | 88.161 | 8 | 16 | Overexpression_connections |
| ccsbBroad304_05982 | *CDX2* | 97.745 | 95.231 | 89.854 | 7 | 7 | Overexpression_connections |
| ccsbBroad304_01579 | *SOX2* | 98.403 | 94.619 | 92.216 | 8 | 16 | Overexpression_connections |
| ccsbBroad304_02451 | *HOXB13* | 96.325 | 91.415 | 82.992 | 8 | 8 | Overexpression_connections |
| ccsbBroad304_07117 | *UGCG* | 95.596 | 91.162 | 84.588 | 7 | 7 | Overexpression_connections |
| ccsbBroad304_06102 | *DLX3* | 96.926 | 90.531 | 73.024 | 8 | 8 | Overexpression_connections |
| ccsbBroad304_06753 | *POU5F1* | 96.32 | 90.492 | 60.328 | 7 | 12 | Overexpression_connections |
| ccsbBroad304_00763 | *HNF4A* | 94.544 | 90.405 | 84.836 | 7 | 12 | Overexpression_connections |
| CGS001-8826 | *IQGAP1* | 99.82 | 99.591 | 99.123 | 9 | 11 | Consensus_Knockdown_connections |
| CGS001-7855 | *FZD5* | 99.92 | 99.47 | 97.85 | 9 | 11 | Consensus_Knockdown_connections |
| CGS001-5245 | *PHB* | 99.469 | 99.298 | 98.382 | 9 | 9 | Consensus_Knockdown_connections |
| CGS001-9063 | *PIAS2* | 99.859 | 98.778 | 89.573 | 9 | 11 | Consensus_Knockdown_connections |
| CGS001-27095 | *TRAPPC3* | 99.639 | 98.776 | 97.867 | 9 | 11 | Consensus_Knockdown_connections |
| CGS001-51290 | *ERGIC2* | 99.797 | 98.749 | 97.547 | 7 | 7 | Consensus_Knockdown_connections |
| CGS001-378 | *ARF4* | 99.366 | 98.713 | 96.82 | 9 | 9 | Consensus_Knockdown_connections |
| CGS001-4793 | *NFKBIB* | 99.012 | 98.592 | 93.988 | 9 | 9 | Consensus_Knockdown_connections |
| CGS001-5329 | *PLAUR* | 99.96 | 98.508 | 61.865 | 6 | 8 | Consensus_Knockdown_connections |
| CGS001-5478 | *PPIA* | 99.192 | 98.484 | 95.663 | 9 | 11 | Consensus_Knockdown_connections |
| CGS001-6697 | *SPR* | 98.91 | 98.387 | 95.954 | 9 | 11 | Consensus_Knockdown_connections |
| CGS001-3725 | *JUN* | 99.258 | 98.34 | 95.76 | 9 | 11 | Consensus_Knockdown_connections |
| CGS001-801 | *CALM1* | 99.222 | 98.237 | 91.761 | 9 | 11 | Consensus_Knockdown_connections |
| CGS001-9612 | *NCOR2* | 99.001 | 97.767 | 94.561 | 8 | 10 | Consensus_Knockdown_connections |
| CGS001-5888 | *RAD51* | 99.248 | 97.734 | 92.931 | 7 | 9 | Consensus_Knockdown_connections |
| CGS001-7405 | *UVRAG* | 98.967 | 97.575 | 95.145 | 8 | 10 | Consensus_Knockdown_connections |
| CGS001-4609 | *MYC* | 98.641 | 97.466 | 94.524 | 9 | 11 | Consensus_Knockdown_connections |
| CGS001-10914 | *PAPOLA* | 98.052 | 97.454 | 94.664 | 9 | 9 | Consensus_Knockdown_connections |
| CGS001-3845 | *KRAS* | 98.718 | 97.401 | 94.497 | 9 | 11 | Consensus_Knockdown_connections |
| CGS001-5608 | *MAP2K6* | 98.173 | 97.218 | 87.303 | 8 | 8 | Consensus_Knockdown_connections |
| CGS001-5045 | *FURIN* | 98.481 | 97.185 | 90.417 | 9 | 11 | Consensus_Knockdown_connections |
| CGS001-4072 | *EPCAM* | 98.618 | 97.173 | 90.901 | 8 | 8 | Consensus_Knockdown_connections |
| CGS001-1184 | *CLCN5* | 98.572 | 97.152 | 88.149 | 7 | 7 | Consensus_Knockdown_connections |
| CGS001-2896 | *GRN* | 99.377 | 97.147 | 92.156 | 9 | 11 | Consensus_Knockdown_connections |
| CGS001-27154 | *BRPF3* | 98.532 | 97.146 | 92.455 | 9 | 9 | Consensus_Knockdown_connections |
| CGS001-2287 | *FKBP3* | 99.348 | 97.054 | 89.178 | 9 | 9 | Consensus_Knockdown_connections |
| CGS001-6832 | *SUPV3L1* | 99.82 | 96.845 | 92.427 | 9 | 11 | Consensus_Knockdown_connections |
| CGS001-7415 | *VCP* | 98.48 | 96.826 | 91.106 | 7 | 7 | Consensus_Knockdown_connections |
| CGS001-90678 | *LRSAM1* | 98.619 | 96.812 | 94.704 | 9 | 9 | Consensus_Knockdown_connections |
| CGS001-27125 | *AFF4* | 98.648 | 96.758 | 90.479 | 8 | 8 | Consensus_Knockdown_connections |
| CGS001-54541 | *DDIT4* | 99.113 | 96.409 | 93.304 | 9 | 11 | Consensus_Knockdown_connections |
| CGS001-10856 | *RUVBL2* | 96.902 | 96.408 | 89.634 | 9 | 9 | Consensus_Knockdown_connections |
| CGS001-8613 | *PPAP2B* | 96.769 | 96.406 | 95.338 | 9 | 9 | Consensus_Knockdown_connections |
| CGS001-51056 | *LAP3* | 98.525 | 96.363 | 91.175 | 9 | 11 | Consensus_Knockdown_connections |
| CGS001-182 | *JAG1* | 98.622 | 96.149 | 91.372 | 9 | 9 | Consensus_Knockdown_connections |
| CGS001-54957 | *TXNL4B* | 98.955 | 96.09 | 89.036 | 9 | 11 | Consensus_Knockdown_connections |
| CGS001-10972 | *TMED10* | 96.856 | 96.038 | 93.591 | 9 | 11 | Consensus_Knockdown_connections |
| CGS001-7263 | *TST* | 96.889 | 95.838 | 84.298 | 9 | 9 | Consensus_Knockdown_connections |
| CGS001-8324 | *FZD7* | 98.953 | 95.687 | 91.453 | 9 | 9 | Consensus_Knockdown_connections |
| CGS001-25989 | *ULK3* | 97.059 | 95.66 | 93.552 | 8 | 8 | Consensus_Knockdown_connections |
| CGS001-55165 | *CEP55* | 98.116 | 95.572 | 90.788 | 9 | 10 | Consensus_Knockdown_connections |
| CGS001-8833 | *GMPS* | 98.366 | 95.52 | 92.443 | 9 | 9 | Consensus_Knockdown_connections |
| CGS001-6256 | *RXRA* | 97.583 | 95.148 | 84.716 | 8 | 10 | Consensus_Knockdown_connections |
| CGS001-5707 | *PSMD1* | 97.775 | 95.12 | 90.122 | 7 | 9 | Consensus_Knockdown_connections |
| CGS001-2355 | *FOSL2* | 98.588 | 94.968 | 87.775 | 8 | 10 | Consensus_Knockdown_connections |
| CGS001-100 | *ADA* | 96.872 | 94.889 | 80.685 | 9 | 11 | Consensus_Knockdown_connections |
| CGS001-5883 | *RAD9A* | 98.606 | 94.882 | 86.109 | 9 | 11 | Consensus_Knockdown_connections |
| CGS001-6129 | *RPL7* | 99.492 | 94.844 | 71.904 | 6 | 8 | Consensus_Knockdown_connections |
| CGS001-21 | *ABCA3* | 99.351 | 94.751 | 77.811 | 7 | 9 | Consensus_Knockdown_connections |
| CGS001-2065 | *ERBB3* | 99.221 | 94.715 | 87.469 | 9 | 11 | Consensus_Knockdown_connections |
| CGS001-10153 | *CEBPZ* | 97.782 | 94.708 | 92.437 | 8 | 9 | Consensus_Knockdown_connections |
| CGS001-527 | *ATP6V0C* | 97.011 | 94.678 | 81.68 | 8 | 8 | Consensus_Knockdown_connections |
| CGS001-5690 | *PSMB2* | 95.512 | 94.643 | 92.462 | 8 | 10 | Consensus_Knockdown_connections |
| CGS001-27020 | *NPTN* | 97.752 | 94.636 | 91.815 | 8 | 8 | Consensus_Knockdown_connections |
| CGS001-1019 | *CDK4* | 98.634 | 94.502 | 81.345 | 9 | 10 | Consensus_Knockdown_connections |
| CGS001-5693 | *PSMB5* | 97.185 | 94.382 | 77.652 | 7 | 7 | Consensus_Knockdown_connections |
| CGS001-388 | *RHOB* | 97.075 | 94.286 | 84.045 | 7 | 7 | Consensus_Knockdown_connections |
| CGS001-340385 | *ZNF517* | 97.72 | 94.276 | 91.009 | 9 | 9 | Consensus_Knockdown_connections |
| CGS001-79073 | *TMEM109* | 97.224 | 94.258 | 91.507 | 8 | 9 | Consensus_Knockdown_connections |
| CGS001-1024 | *CDK8* | 98.581 | 94.249 | 87.517 | 9 | 12 | Consensus_Knockdown_connections |
| CGS001-55837 | *EAPP* | 95.941 | 94.071 | 85.323 | 8 | 9 | Consensus_Knockdown_connections |
| CGS001-2618 | *GART* | 99.108 | 93.666 | 86.1 | 8 | 8 | Consensus_Knockdown_connections |
| CGS001-836 | *CASP3* | 99.371 | 93.648 | 88.907 | 9 | 11 | Consensus_Knockdown_connections |
| CGS001-523 | *ATP6V1A* | 98.017 | 93.629 | 86.605 | 9 | 9 | Consensus_Knockdown_connections |
| CGS001-5664 | *PSEN2* | 96.875 | 93.578 | 83.392 | 7 | 7 | Consensus_Knockdown_connections |
| CGS001-6389 | *SDHA* | 96.7 | 93.572 | 83.479 | 9 | 9 | Consensus_Knockdown_connections |
| CGS001-3198 | *HOXA1* | 96.731 | 93.549 | 86.845 | 9 | 9 | Consensus_Knockdown_connections |
| CGS001-644 | *BLVRA* | 97.939 | 93.47 | 82.038 | 9 | 11 | Consensus_Knockdown_connections |
| CGS001-9123 | *SLC16A3* | 95.43 | 93.466 | 75.296 | 8 | 8 | Consensus_Knockdown_connections |
| CGS001-57448 | *BIRC6* | 96.771 | 93.43 | 90.316 | 9 | 9 | Consensus_Knockdown_connections |
| CGS001-3980 | *LIG3* | 96.583 | 93.403 | 76.3 | 7 | 7 | Consensus_Knockdown_connections |
| CGS001-2664 | *GDI1* | 94.838 | 93.303 | 88.731 | 8 | 8 | Consensus_Knockdown_connections |
| CGS001-10253 | *SPRY2* | 96.568 | 93.278 | 72.074 | 8 | 8 | Consensus_Knockdown_connections |
| CGS001-5170 | *PDPK1* | 98.624 | 93.216 | 86.894 | 9 | 11 | Consensus_Knockdown_connections |
| CGS001-545 | *ATR* | 97.594 | 93.199 | 89.385 | 9 | 12 | Consensus_Knockdown_connections |
| CGS001-23476 | *BRD4* | 96.315 | 93.193 | 83.132 | 8 | 10 | Consensus_Knockdown_connections |
| CGS001-58495 | *OVOL2* | 97.369 | 93.087 | 80.532 | 7 | 7 | Consensus_Knockdown_connections |
| CGS001-63027 | *SLC22A23* | 97.649 | 92.837 | 61.891 | 8 | 8 | Consensus_Knockdown_connections |
| CGS001-51231 | *VRK3* | 95.84 | 92.776 | 80.216 | 9 | 11 | Consensus_Knockdown_connections |
| CGS001-10325 | *RRAGB* | 95.969 | 92.741 | 73.375 | 8 | 8 | Consensus_Knockdown_connections |
| CGS001-9831 | *ZNF623* | 94.634 | 92.731 | 80.449 | 9 | 9 | Consensus_Knockdown_connections |
| CGS001-9924 | *PAN2* | 95.449 | 92.585 | 87.115 | 9 | 11 | Consensus_Knockdown_connections |
| CGS001-10682 | *EBP* | 96.255 | 92.558 | 89.131 | 9 | 11 | Consensus_Knockdown_connections |
| CGS001-7251 | *TSG101* | 98.576 | 92.507 | 85.365 | 8 | 8 | Consensus_Knockdown_connections |
| CGS001-6208 | *RPS14* | 99.001 | 92.504 | 84.332 | 8 | 8 | Consensus_Knockdown_connections |
| CGS001-26574 | *AATF* | 96.053 | 92.464 | 77.771 | 8 | 10 | Consensus_Knockdown_connections |
| CGS001-6778 | *STAT6* | 95.078 | 92.454 | 89.417 | 9 | 11 | Consensus_Knockdown_connections |
| CGS001-3398 | *ID2* | 98.479 | 92.382 | 84.874 | 9 | 11 | Consensus_Knockdown_connections |
| CGS001-10465 | *PPIH* | 95.264 | 92.362 | 76.15 | 8 | 8 | Consensus_Knockdown_connections |
| CGS001-6714 | *SRC* | 95.8 | 92.318 | 85.012 | 9 | 11 | Consensus_Knockdown_connections |
| CGS001-8766 | *RAB11A* | 97.213 | 92.284 | 82.865 | 9 | 9 | Consensus_Knockdown_connections |
| CGS001-6203 | *RPS9* | 96.673 | 92.247 | 80.852 | 8 | 8 | Consensus_Knockdown_connections |
| CGS001-6647 | *SOD1* | 96.467 | 92.24 | 78.671 | 9 | 12 | Consensus_Knockdown_connections |
| CGS001-25966 | *C2CD2* | 93.637 | 92.157 | 74.15 | 8 | 9 | Consensus_Knockdown_connections |
| CGS001-760 | *CA2* | 95.385 | 92.055 | 85.707 | 8 | 10 | Consensus_Knockdown_connections |
| CGS001-1717 | *DHCR7* | 94.639 | 92.05 | 86.887 | 7 | 9 | Consensus_Knockdown_connections |
| CGS001-1633 | *DCK* | 94.409 | 91.998 | 81.459 | 9 | 11 | Consensus_Knockdown_connections |
| CGS001-226 | *ALDOA* | 95.262 | 91.981 | 83.517 | 7 | 7 | Consensus_Knockdown_connections |
| CGS001-51274 | *KLF3* | 95.539 | 91.913 | 74.864 | 9 | 9 | Consensus_Knockdown_connections |
| CGS001-7272 | *TTK* | 98.033 | 91.891 | 78.251 | 8 | 10 | Consensus_Knockdown_connections |
| CGS001-5695 | *PSMB7* | 95.667 | 91.866 | 86.441 | 8 | 8 | Consensus_Knockdown_connections |
| CGS001-84669 | *USP32* | 97.542 | 91.759 | 74.989 | 9 | 9 | Consensus_Knockdown_connections |
| CGS001-5927 | *KDM5A* | 93.993 | 91.735 | 85.322 | 9 | 11 | Consensus_Knockdown_connections |
| CGS001-10450 | *PPIE* | 95.198 | 91.632 | 87.259 | 9 | 11 | Consensus_Knockdown_connections |
| CGS001-51053 | *GMNN* | 96.133 | 91.543 | 79.176 | 9 | 9 | Consensus_Knockdown_connections |
| CGS001-8751 | *ADAM15* | 94.484 | 91.534 | 75.768 | 9 | 11 | Consensus_Knockdown_connections |
| CGS001-2114 | *ETS2* | 96.461 | 91.478 | 88.806 | 9 | 11 | Consensus_Knockdown_connections |
| CGS001-255738 | *PCSK9* | 95.442 | 91.441 | 76.377 | 8 | 8 | Consensus_Knockdown_connections |
| CGS001-7541 | *ZFP161* | 93.88 | 91.433 | 67.204 | 8 | 9 | Consensus_Knockdown_connections |
| CGS001-6194 | *RPS6* | 95.772 | 91.315 | 86.271 | 9 | 11 | Consensus_Knockdown_connections |
| CGS001-7453 | *WARS* | 98.006 | 91.222 | 82.188 | 9 | 9 | Consensus_Knockdown_connections |
| CGS001-1173 | *AP2M1* | 97.676 | 91.218 | 69.754 | 8 | 8 | Consensus_Knockdown_connections |
| CGS001-2146 | *EZH2* | 95.371 | 91.138 | 88.603 | 9 | 12 | Consensus_Knockdown_connections |
| CGS001-23466 | *CBX6* | 94.088 | 91.12 | 80.447 | 8 | 8 | Consensus_Knockdown_connections |
| CGS001-7316 | *UBC* | 98.91 | 91.04 | 76.276 | 9 | 9 | Consensus_Knockdown_connections |
| CGS001-5209 | *PFKFB3* | 94.441 | 91.032 | 80.975 | 9 | 9 | Consensus_Knockdown_connections |
| CGS001-1642 | *DDB1* | 94.404 | 90.995 | 69.242 | 8 | 8 | Consensus_Knockdown_connections |
| CGS001-386724 | *AMIGO3* | 96.042 | 90.922 | 88.003 | 9 | 11 | Consensus_Knockdown_connections |
| CGS001-5747 | *PTK2* | 98.181 | 90.905 | 86.831 | 9 | 12 | Consensus_Knockdown_connections |
| CGS001-6566 | *SLC16A1* | 97.58 | 90.794 | 66.792 | 8 | 10 | Consensus_Knockdown_connections |
| CGS001-58472 | *SQRDL* | 97.922 | 90.742 | 83.247 | 9 | 10 | Consensus_Knockdown_connections |
| CGS001-4172 | *MCM3* | 94.36 | 90.682 | 84.787 | 9 | 11 | Consensus_Knockdown_connections |
| CGS001-5091 | *PC* | 96.977 | 90.655 | 82.295 | 9 | 9 | Consensus_Knockdown_connections |
| CGS001-3572 | *IL6ST* | 94.736 | 90.588 | 86.608 | 9 | 10 | Consensus_Knockdown_connections |
| CGS001-7167 | *TPI1* | 96.156 | 90.577 | 68.991 | 7 | 9 | Consensus_Knockdown_connections |
| CGS001-25799 | *ZNF324* | 94.179 | 90.554 | 87.228 | 8 | 8 | Consensus_Knockdown_connections |
| CGS001-2821 | *GPI* | 98.169 | 90.491 | 65.655 | 8 | 8 | Consensus_Knockdown_connections |
| CGS001-23075 | *SWAP70* | 95.009 | 90.457 | 87.466 | 9 | 11 | Consensus_Knockdown_connections |
| CGS001-355 | *FAS* | 95.166 | 90.45 | 84.707 | 9 | 11 | Consensus_Knockdown_connections |
| CGS001-572 | *BAD* | 98.19 | 90.363 | 76.355 | 9 | 11 | Consensus_Knockdown_connections |
| CGS001-178 | *AGL* | 94.499 | 90.338 | 85.364 | 9 | 11 | Consensus_Knockdown_connections |
| CGS001-30836 | *DNTTIP2* | 99.15 | 90.299 | 83.008 | 9 | 10 | Consensus_Knockdown_connections |
| CGS001-6499 | *SKIV2L* | 97.552 | 90.289 | 65.853 | 9 | 11 | Consensus_Knockdown_connections |
| CGS001-6259 | *RYK* | 94.566 | 90.217 | 83.314 | 8 | 10 | Consensus_Knockdown_connections |
| CGS001-57547 | *ZNF624* | 98.536 | 90.177 | 77.668 | 8 | 8 | Consensus_Knockdown_connections |
| CGS001-23443 | *SLC35A3* | 92.191 | 90.102 | 83.179 | 9 | 11 | Consensus_Knockdown_connections |
| CGS001-10475 | *TRIM38* | 97.947 | 90.088 | 82.743 | 8 | 9 | Consensus_Knockdown_connections |
| CGS001-7083 | *TK1* | 95.284 | 90.006 | 60.004 | 8 | 8 | Consensus_Knockdown_connections |

**Supplementary Table 12. KEGG pathway enrichment of cellular turnover-correlating genes with a LINCS signature.**

The bootstrap value is defined as the number of times (out of 5,000) that *P*-values of a random gene list were smaller than the *P*-value of each gene set.

| **Pathway Name** | **Count** | **Pathway**  **Size** | **Adj**  ***P*-value** | **Bootstrap**  ***P*-value** | **genes** |
| --- | --- | --- | --- | --- | --- |
| KEGG_ERBB_SIGNALING_PATHWAY | 9 | 87 | 1.3E-03 | 0.0E+00 | *JUN,BAD,PTK2,ERBB3,MYC,KRAS,CDKN1B,CDKN1A,SRC* |
| KEGG_PATHWAYS_IN_CANCER | 15 | 324 | 1.1E-02 | 0.0E+00 | *JUN,FZD7,PTK2,MYC,KRAS,BAD,CASP3,PIAS2,FZD5,RAD51,RXRA,CDKN1B,CDKN1A,CDK4,FAS* |
| KEGG_CELL_CYCLE | 8 | 124 | 4.0E-02 | 2.0E-04 | *ATR,MYC,MCM3,CDKN1B,CDKN1A,CDKN2C,TTK,CDK4* |
| KEGG_CHRONIC_MYELOID_LEUKEMIA | 6 | 73 | 4.2E-02 | 2.0E-04 | *BAD,MYC,KRAS,CDKN1B,CDKN1A,CDK4* |

**Supplementary Table 13. Biological process (BP) Gene Ontology (GO) term enrichment of cellular turnover-correlating genes with a LINCS signature.**

**The bootstrap value is defined as the number of times (out of 5,000) that *P*-values of a random gene list were smaller than the *P*-value of each gene set.**

| **Gene Ontology (GO) term** | **Count** | **GO term**  **size** | **Adj**  ***P*-value** | **Bootstrap**  ***P-*value** | **genes** |
| --- | --- | --- | --- | --- | --- |
| DNA_REPAIR | 9 | 123 | 3.9E-02 | 0.0E+00 | *LIG3,RAD9A,ATR,VCP,UVRAG,DDB1,RAD51,RUVBL2,SOD1* |
| RESPONSE_TO_DNA_DAMAGE_STIMULUS | 10 | 159 | 3.9E-02 | 0.0E+00 | *MAP2K6,LIG3,RAD9A,ATR,VCP,UVRAG,DDB1,RAD51,RUVBL2,SOD1* |
| RESPONSE_TO_ENDOGENOUS_STIMULUS | 11 | 196 | 3.9E-02 | 0.0E+00 | *MAP2K6,LIG3,RAD9A,ATR,UVRAG,RAD51,RUVBL2,PCSK9,SOD1,VCP,DDB1* |
| CELL_CYCLE_GO_0007049 | 14 | 313 | 3.9E-02 | 0.0E+00 | *MAP2K6,LIG3,RAD9A,ATR,MYC,RAD51,GMNN,TTK,CDKN2C,CDKN1A,CDKN1B,RHOB,DDB1,CDK4* |
| REGULATION_OF_CELL_CYCLE | 10 | 180 | 5.0E-02 | 0.0E+00 | *TTK,CDKN2C,MAP2K6,RAD9A,ATR,CDKN1B,RHOB,MYC,DDB1,GMNN* |
| DNA_METABOLIC_PROCESS | 12 | 255 | 5.0E-02 | 0.0E+00 | *LIG3,RAD9A,SUPV3L1,ATR,UVRAG,RAD51,RUVBL2,GMNN,SOD1,VCP,DDB1,MCM3* |

**SUPPLEMENTARY RESULTS AND DISCUSSION**

**Taking into account the relatedness of human cells and tissues**

Both simple linear regression and standard Analysis of Variance (ANOVA) typically require errors to be statistically independent of one another. However, in our dataset the cells and tissues formed distinctive expression clusters (Figure 1), indicating that assumption of sample independence may potentially be violated. To account for this relatedness, we applied phylogenetic ANOVA and generalized least square regression, which have been used in cross-species evolutionary studies to account for the effect of phylogeny[^33-36^](#_ENREF_33). In phylogenetic ANOVA, the F-value of standard ANOVA is compared to a null distribution generated by simulating trait evolution on a reference tree, so that only those genes that exhibit between-group differences greater than expected based on the inherent hierarchical structure will be reported as significant.

**Differential gene expression in heart, thyroid, and hematopoietic tissues and monocytes**

Genes differentially expressed (phylogenetic ANOVA *P*-value ≤ 0.01) in the heart alone (Supplementary Figure 2A) included genes critical for heart function such as the vasodilator atrial natriuretic peptide (*NPPA*)[^39^](#_ENREF_39), cardiac sodium channel Nav1.5 (*SCN5A*)[^40^](#_ENREF_40) and the AMPK subunit encoding *PRKGA2*[^41^](#_ENREF_41), while thyroid gland showed increased expression of a G-protein α subunit (*GNAS*) required for thyroid hormone receptor signaling[^42^](#_ENREF_42), vascular endothelial growth factor A (*VEGFA*)[^43^](#_ENREF_43) and the thyroid transcription factor PAX8 (*PAX8*)[^44^](#_ENREF_44) (Supplementary Figure 2B).

On the other hand, 605 genes (out of 12,044) showed significant differential expression in the hematopoietic tissues, bone marrow and spleen, and monocytes (Supplementary Figure 2C and Supplementary Table 3). These included several immune-related genes (Supplementary Table 3), which is consistent with a recent study demonstrating that blood and spleen have highly similar transcriptomes due to a large number of monocytes residing in the spleen before being recruited to damaged cells and tissues[^45^](#_ENREF_45).

**Expression of the complex SNRPN-SNURF locus positively correlates with cellular turnover**

*SNRPN* (small nuclear ribonucleoprotein polypeptide N) and *SNURF* (SNRPN upstream reading frame) were positively correlated with turnover (Supplementary Table 5 and Supplementary Figure 3). They are annotated as two distinct protein-coding genes but are derived from a complex locus on chromosome 15, *SNURF–SNRPN,* transcribing a bicistronic RNA[^46^](#_ENREF_46). The expression of *SNURF* and *SNRPN* largely overlapped (Supplementary Figure 3); however, SNURF was usually expressed at a lower level, which likely stems from RNA-seq reads spanning its shorter coding sequence compared to *SNRPN* (71 amino acids *vs* 240 amino acids)[^46^](#_ENREF_46). *SNURF–SNRPN* mRNA is reported to be highly expressed in brain and heart[^46^](#_ENREF_46), in agreement with our data (high expression in neuron and heart muscle).

The function of the SNURF protein is not well established; however, evidence is emerging that the *SNRPN*-encoded small nuclear ribonucleoprotein polypeptide N (SmN) regulates alternative splicing[^47^](#_ENREF_47). In addition to SNURF and SmN proteins, the *SNURF–SNRPN* locus also gives rise to a number of intron-derived non-coding RNAs termed small nucleolar RNAs (snoRNAs)[^48^](#_ENREF_48). Interestingly, the *SNURF–SNRPN* promoter is inactivated in many Prader-Willi syndrome (PWS) subjects, which present premature aging symptoms including cardiovascular disease and dementia. While the *SNURF–SNRPN* locus has not been directly linked to classical PWS phenotypes such as hyperphagia[^50^](#_ENREF_50), we speculate that precise regulation and expression of the complex *SNURF–SNRPN* locus, which can give rise to two distinct proteins and a number of non-coding RNAs, may contribute to healthy aging.

**SUPPLEMENTARY EXPERIMENTAL PROCEDURES**

# Sample considerations

Obtaining accurate cell and tissue turnover data from the literature is challenging due to technical and methodological obstacles. Turnover measurements of individual cell types are generally lacking. Notable exceptions include neurons[^14^](#_ENREF_14) and cells with well-established short lifespans such as monocytes and hepatocytes. Recently, ^14^C radiocarbon dating of DNA has been utilized to validate tissue turnover; this method is usually restricted to tissues or large cell preparations because very little ^14^C is incorporated into single cells. However, cell lifespan at the level of tissue (cell turnover time for a particular tissue) data must be approached with care. Many cell types show great diversity in turnover among individual cells in the lineage. For example, gut epithelial lifespan varies considerably, from days to a decade, depending on whether cell populations sampled comprise the gastrointestinal lining or not[^1^](#_ENREF_1). Another challenge with gene expression analyses is the fact that single-cell RNA-sequencing (RNA-seq) is yet to gain widespread usage[^55^](#_ENREF_55), which largely limits current analyses to tissues. Taking the above limitations into account, we restricted our transcriptome analysis to 21 adult somatic cells and tissues with multiple biological replicates and cellular turnover as *bona fide* cellular lifespan estimates[^6^](#_ENREF_6), which we supplemented with additional data collected through primary literature searches (Supplementary Tables 1 and 2).

**Gene expression data processing**

Publicly-available Illumina transcriptome (RNA-seq) data (summarized in Supplementary Table 1; individual sample information in Supplementary Table 2), representing human adult somatic tissue or cell types, were obtained from EBI ArrayExpress[^56^](#_ENREF_56), the NCBI Short Read Archive (SRA)[^57^](#_ENREF_57), and the ENCODE (Encyclopedia of DNA Elements) web site (https://www.encodeproject.org). For consistency, all RNA-seq data sets were from paired-end libraries. FASTQ files were aligned to the human genome, UCSC build hg19 downloaded from the Illumina iGenome resource at http://goo.gl/LspLBZ, using the spliced-read mapper TopHat (v2.0.9)[^58^](#_ENREF_58) and reference gene annotations to guide the alignment. Raw gene counts were computed from TopHat-generated BAM files and reference gene annotations using featureCounts v1.4.5-p1, counting coding sequence (CDS) features of the UCSC hg19 gene annotation file (gtf)[^59^](#_ENREF_59). Raw RNA-seq counts were scaled using the quantile normalization method[^60^](#_ENREF_60) available in the R package ‘preprocessCore’. Genes with a quantile-normalized count of at least 1.0 in all sample types were retained, yielding 12,044 genes. Principal components analysis was performed using the R package ‘scatterplot3d’[^61^](#_ENREF_61). Pair-wise correlations (correlograms) were calculated and visualized as a correlation matrix using the R package ‘corrplot’.

**Gene expression tree**

A neighbor-joining (NJ)[^62^](#_ENREF_62) gene expression tree was constructed using the R packages ’supraHex’[^63^](#_ENREF_63) (visTreeBootstrap function) and ‘ape’[^64^](#_ENREF_64), with one million bootstrap replications to test relative nodal support in the topology. Bootstrap values refer to proportions of replicate trees that share the same branching pattern of the consensus tree.

**Phylogenetic ANOVA**

To reveal cell- or tissue-specific gene expression we performed phylogenetic ANOVA, which controls for tissue autocorrelation, using the phy.anova function implemented in the R package ‘geiger’[^65^](#_ENREF_65) with average log transformed gene expression values from each cell or tissue and setting simulations to 1,000.

**Identification of gene expression correlating with cellular turnover by generalized least squares regression**

Generalized least squares (GLS) regression[^66^](#_ENREF_66) was performed to identify genes whose expression correlates with turnover. Briefly, normalized counts and estimated turnover (days) were log transformed to satisfy model assumptions of normally distributed residuals (homoscedacity). The age of human donors was not available for a number of cell and tissue types and ‘age’ was excluded in the regression; however, the mean and median age of samples in the data set was approximately 56 years (Supplementary Table 1). To minimize stochastic variability between cells of the same age and type, we employed the average gene expression count of each sample type in our analysis. The R package ‘ape’[^64^](#_ENREF_64) was used for tree handling and ‘nlme’[^69^](#_ENREF_69) for regression modeling. We considered the following models in the regression: the NULL model (i.e. simple linear regression, assuming constant variance and independent errors) and classic models for continuous transcriptome evolution, the Brownian motion (BM) and Ornstein-Uhlenbeck (OU) models (for OU, the restraining force, α, was set to 1.0 and 2.0). The Brownian model describes neutral evolution of gene expression[^34^](#_ENREF_34) whereas the OU model is often used for stabilizing selection. For each gene, the best-fit model was selected based on maximum likelihood (ML) scores. Likelihood solutions can be unstable, especially when the number of data points per parameters is less than ~10 [^72^](#_ENREF_72), thus we restricted our analysis to the cells and tissues from various germ cell lineages rather than comparing distinct germ layer lineages (e.g. only 7 cells and tissues were derived from the ectoderm).

**Gene set enrichment analysis (GSEA)**

Curated Gene Ontology (GO) and KEGG pathway gene sets were downloaded from MSigDB (http://www.broadinstitute.org/gsea/msigdb/collections.jsp#C1)[^73^](#_ENREF_73) and assessed using a custom R script. Statistical significance of gene enrichment results was assessed by bootstrapping[^74^](#_ENREF_74), with 5,000 sample permutations (randomly shuffling the vector of gene expression values for each gene). The bootstrap value is defined as the number of times (out of 5,000) that *P*-values of the random gene list were smaller than the *P*-value of each gene set. Visualization of enriched pathways was performed by interrogating the manually-curated database INOH (Integrating Network Objects with Hierarchies)[^75^](#_ENREF_75) via the InnateDB resource[^76^](#_ENREF_76). Protein-protein interactions were identified using STRING (Search Tool for the Retrieval of Interacting Genes/Proteins)[^77^](#_ENREF_77), with default parameters. The Library of Integrated Network-based Cellular Signatures (LINCS), a database of gene expression profiles from human cells treated with small molecules and genetic (small hairpin-mediated knockdown, shRNA, and gene overexpression) perturbations (http://www.lincscloud.org), was queried with genes whose expression correlated with cellular turnover. The release of LINCS (vA2) contained 476,251 signatures derived from 1,328,098 experiments.

**SUPPLEMENTARY REFERENCES**

1. Vickaryous, M.K. & Hall, B.K. Human cell type diversity, evolution, development, and classification with special reference to cells derived from the neural crest. *Biol Rev Camb Philos Soc* **81**, 425-55 (2006).

2. Strawford, A., Antelo, F., Christiansen, M. & Hellerstein, M.K. Adipose tissue triglyceride turnover, de novo lipogenesis, and cell proliferation in humans measured with 2H2O. *Am J Physiol Endocrinol Metab* **286**, E577-88 (2004).

3. Spalding, K.L. *et al.* Dynamics of fat cell turnover in humans. *Nature* **453**, 783-7 (2008).

4. Arner, E. *et al.* Adipocyte turnover: relevance to human adipose tissue morphology. *Diabetes* **59**, 105-9 (2010).

5. Leblond, C.P. Classification of Cell Populations on the Basis of Their Proliferative Behavior. *Natl Cancer Inst Monogr* **14**, 119-50 (1964).

6. Richardson, R.B., Allan, D.S. & Le, Y. Greater organ involution in highly proliferative tissues associated with the early onset and acceleration of ageing in humans. *Exp Gerontol* **55**, 80-91 (2014).

7. Fahy, R.J., Doseff, A.I. & Wewers, M.D. Spontaneous human monocyte apoptosis utilizes a caspase-3-dependent pathway that is blocked by endotoxin and is independent of caspase-1. *J Immunol* **163**, 1755-62 (1999).

8. Mangan, D.F. & Wahl, S.M. Differential regulation of human monocyte programmed cell death (apoptosis) by chemotactic factors and pro-inflammatory cytokines. *J Immunol* **147**, 3408-12 (1991).

9. Cole, J.W. & Mc, K.A. Observations of cell renewal in human rectal mucosa in vivo with thymidine-H3. *Gastroenterology* **41**, 122-5 (1961).

10. Lipkin, M. Cell Replication in the Gastrointestinal Tract of Man. *Gastroenterology* **48**, 616-24 (1965).

11. Gurbuz, B., Yalti, S. & Yildirim, G. Endometrial thickness and uterine size in postmenopausal women. *Int J Gynaecol Obstet* **84**, 268-70 (2004).

12. Amir, W. *et al.* Predicting factors for endometrial thickness during treatment with assisted reproductive technology. *Fertil Steril* **87**, 799-804 (2007).

13. Bertalanffy, F.D. Tritiated Thymidine Versus Colchicine Technique in the Study of Cell Population Cytodynamics. *Lab Invest* **13**, 871-86 (1964).

14. Bergmann, O. *et al.* Evidence for cardiomyocyte renewal in humans. *Science* **324**, 98-102 (2009).

15. Bergmann, O. *et al.* Dynamics of Cell Generation and Turnover in the Human Heart. *Cell* **161**, 1566-75 (2015).

16. Halprin, K.M. Epidermal "turnover time"--a re-examination. *Br J Dermatol* **86**, 14-9 (1972).

17. Zajicek, G., Oren, R. & Weinreb, M., Jr. The streaming liver. *Liver* **5**, 293-300 (1985).

18. Blenkinsopp, W.K. Proliferation of respiratory tract epithelium in the rat. *Exp Cell Res* **46**, 144-54 (1967).

19. Spalding, K.L., Bhardwaj, R.D., Buchholz, B.A., Druid, H. & Frisen, J. Retrospective birth dating of cells in humans. *Cell* **122**, 133-43 (2005).

20. Huttner, H.B. *et al.* The age and genomic integrity of neurons after cortical stroke in humans. *Nat Neurosci* **17**, 801-3 (2014).

21. Jilka, R.L. *et al.* Increased bone formation by prevention of osteoblast apoptosis with parathyroid hormone. *J Clin Invest* **104**, 439-46 (1999).

22. Jahn, K., Richards, R.G., Archer, C.W. & Stoddart, M.J. Pellet culture model for human primary osteoblasts. *Eur Cell Mater* **20**, 149-61 (2010).

23. Okumura, K., Shinohara, M. & Endo, F. Capability of tissue stem cells to organize into salivary rudiments. *Stem Cells Int* **2012**, 502136 (2012).

24. Lindholm, M.E. *et al.* The human skeletal muscle transcriptome: sex differences, alternative splicing, and tissue homogeneity assessed with RNA sequencing. *FASEB J* **28**, 4571-81 (2014).

25. Klinger, R.Y., Blum, J.L., Hearn, B., Lebow, B. & Niklason, L.E. Relevance and safety of telomerase for human tissue engineering. *Proc Natl Acad Sci U S A* **103**, 2500-5 (2006).

26. Ho, C., van der Veer, E., Akawi, O. & Pickering, J.G. SIRT1 markedly extends replicative lifespan if the NAD+ salvage pathway is enhanced. *FEBS Lett* **583**, 3081-5 (2009).

27. Andreasen, E. & Ottesen, J. Studies on the lymphocyte production. Investigations on the nucleic acid turnover in the lymphoid organs. *Acta Physiologica Scandinavica* **10**, 258-270 (1945).

28. Coclet, J., Foureau, F., Ketelbant, P., Galand, P. & Dumont, J.E. Cell population kinetics in dog and human adult thyroid. *Clin Endocrinol (Oxf)* **31**, 655-65 (1989).

29. Leblond, C.P. & Walker, B.E. Renewal of cell populations. *Physiol Rev* **36**, 255-76 (1956).

30. Fagerberg, L. *et al.* Analysis of the human tissue-specific expression by genome-wide integration of transcriptomics and antibody-based proteomics. *Mol Cell Proteomics* **13**, 397-406 (2014).

31. Uhlen, M. *et al.* Proteomics. Tissue-based map of the human proteome. *Science* **347**, 1260419 (2015).

32. ENCODE Project Consortium. An integrated encyclopedia of DNA elements in the human genome. *Nature* **489**, 57-74 (2012).

33. Blomberg, S.P., Garland, T., Jr. & Ives, A.R. Testing for phylogenetic signal in comparative data: behavioral traits are more labile. *Evolution* **57**, 717-45 (2003).

34. Felsenstein, J. Phylogenies and the comparative method. *American Naturalist*, 1-15 (1985).

35. Martins, E.P. & Hansen, T.F. Phylogenies and the comparative method: a general approach to incorporating phylogenetic information into the analysis of interspecific data. *American Naturalist*, 646-667 (1997).

36. Pagel, M. Inferring the historical patterns of biological evolution. *Nature* **401**, 877-84 (1999).

37. Garland, T., Dickerman, A.W., Janis, C.M. & Jones, J.A. Phylogenetic analysis of covariance by computer simulation. *Systematic Biology* **42**, 265-292 (1993).

38. Garland, T., Jr., Bennett, A.F. & Rezende, E.L. Phylogenetic approaches in comparative physiology. *J Exp Biol* **208**, 3015-35 (2005).

39. Del Ry, S., Cabiati, M. & Clerico, A. Natriuretic peptide system and the heart. *Front Horm Res* **43**, 134-43 (2014).

40. Liu, M., Yang, K.C. & Dudley, S.C., Jr. Cardiac sodium channel mutations: why so many phenotypes? *Nat Rev Cardiol* **11**, 607-15 (2014).

41. Zaha, V.G. & Young, L.H. AMP-activated protein kinase regulation and biological actions in the heart. *Circ Res* **111**, 800-14 (2012).

42. Weinstein, L.S., Liu, J., Sakamoto, A., Xie, T. & Chen, M. Minireview: GNAS: normal and abnormal functions. *Endocrinology* **145**, 5459-64 (2004).

43. Sato, K. *et al.* Stimulation by thyroid-stimulating hormone and Grave's immunoglobulin G of vascular endothelial growth factor mRNA expression in human thyroid follicles in vitro and flt mRNA expression in the rat thyroid in vivo. *J Clin Invest* **96**, 1295-302 (1995).

44. Mansouri, A., Chowdhury, K. & Gruss, P. Follicular cells of the thyroid gland require Pax8 gene function. *Nat Genet* **19**, 87-90 (1998).

45. Swirski, F.K. *et al.* Identification of splenic reservoir monocytes and their deployment to inflammatory sites. *Science* **325**, 612-6 (2009).

46. Gray, T.A., Saitoh, S. & Nicholls, R.D. An imprinted, mammalian bicistronic transcript encodes two independent proteins. *Proc Natl Acad Sci U S A* **96**, 5616-21 (1999).

47. Lee, M.S. *et al.* Modulation of alternative splicing by expression of small nuclear ribonucleoprotein polypeptide N. *FEBS J* **281**, 5194-207 (2014).

48. Yin, Q.F. *et al.* Long noncoding RNAs with snoRNA ends. *Mol Cell* **48**, 219-30 (2012).

49. Maina, E.N. *et al.* Analysis of candidate imprinted genes in PWS subjects with atypical genetics: a possible inactivating mutation in the SNURF/SNRPN minimal promoter. *J Hum Genet* **52**, 297-307 (2007).

50. Sahoo, T. *et al.* Prader-Willi phenotype caused by paternal deficiency for the HBII-85 C/D box small nucleolar RNA cluster. *Nat Genet* **40**, 719-21 (2008).

51. Sinnema, M., Schrander-Stumpel, C.T., Maaskant, M.A., Boer, H. & Curfs, L.M. Aging in Prader-Willi syndrome: twelve persons over the age of 50 years. *Am J Med Genet A* **158A**, 1326-36 (2012).

52. Whittington, J.E., Holland, A.J. & Webb, T. Ageing in people with Prader-Willi syndrome: mortality in the UK population cohort and morbidity in an older sample of adults. *Psychol Med*, 1-7 (2014).

53. Macdonald, R.A. "Lifespan" of liver cells. Autoradio-graphic study using tritiated thymidine in normal, cirrhotic, and partially hepatectomized rats. *Arch Intern Med* **107**, 335-43 (1961).

54. Perl, S. *et al.* Significant human beta-cell turnover is limited to the first three decades of life as determined by in vivo thymidine analog incorporation and radiocarbon dating. *J Clin Endocrinol Metab* **95**, E234-9 (2010).

55. Shapiro, E., Biezuner, T. & Linnarsson, S. Single-cell sequencing-based technologies will revolutionize whole-organism science. *Nat Rev Genet* **14**, 618-30 (2013).

56. Kolesnikov, N. *et al.* ArrayExpress update--simplifying data submissions. *Nucleic Acids Res* **43**, D1113-6 (2015).

57. Wheeler, D.L. *et al.* Database resources of the National Center for Biotechnology Information. *Nucleic Acids Res* **36**, D13-21 (2008).

58. Kim, D. *et al.* TopHat2: accurate alignment of transcriptomes in the presence of insertions, deletions and gene fusions. *Genome Biol* **14**, R36 (2013).

59. Liao, Y., Smyth, G.K. & Shi, W. featureCounts: an efficient general purpose program for assigning sequence reads to genomic features. *Bioinformatics* **30**, 923-30 (2014).

60. Yue, F. *et al.* A comparative encyclopedia of DNA elements in the mouse genome. *Nature* **515**, 355-64 (2014).

61. Ligges, U. & Mächler, M. Scatterplot3d-an r package for visualizing multivariate data. (Technical Report, SFB 475: Komplexitätsreduktion in Multivariaten Datenstrukturen, Universität Dortmund, 2002).

62. Saitou, N. & Nei, M. The neighbor-joining method: a new method for reconstructing phylogenetic trees. *Mol Biol Evol* **4**, 406-25 (1987).

63. Fang, H. & Gough, J. supraHex: an R/Bioconductor package for tabular omics data analysis using a supra-hexagonal map. *Biochem Biophys Res Commun* **443**, 285-9 (2014).

64. Paradis, E., Claude, J. & Strimmer, K. APE: Analyses of Phylogenetics and Evolution in R language. *Bioinformatics* **20**, 289-90 (2004).

65. Harmon, L.J., Weir, J.T., Brock, C.D., Glor, R.E. & Challenger, W. GEIGER: investigating evolutionary radiations. *Bioinformatics* **24**, 129-31 (2008).

66. Rohlf, F.J. A comment on phylogenetic correction. *Evolution* **60**, 1509-15 (2006).

67. Herndon, L.A. *et al.* Stochastic and genetic factors influence tissue-specific decline in ageing C. elegans. *Nature* **419**, 808-14 (2002).

68. Mele, M. *et al.* Human genomics. The human transcriptome across tissues and individuals. *Science* **348**, 660-5 (2015).

69. Pinheiro, J., Bates, D., DebRoy, S. & Sarkar, D. Linear and nonlinear mixed effects models. *R package version* **3**, 57 (2007).

70. Bedford, T. & Hartl, D.L. Optimization of gene expression by natural selection. *Proc Natl Acad Sci U S A* **106**, 1133-8 (2009).

71. Brawand, D. *et al.* The evolution of gene expression levels in mammalian organs. *Nature* **478**, 343-8 (2011).

72. Pagel, M. User’s manual for Continuous. (2007).

73. Subramanian, A. *et al.* Gene set enrichment analysis: a knowledge-based approach for interpreting genome-wide expression profiles. *Proc Natl Acad Sci U S A* **102**, 15545-50 (2005).

74. Efron, B. & Tibshirani, R. Bootstrap methods for standard errors, confidence intervals, and other measures of statistical accuracy. *Statistical science*, 54-75 (1986).

75. Yamamoto, S. *et al.* INOH: ontology-based highly structured database of signal transduction pathways. *Database (Oxford)* **2011**, bar052 (2011).

76. Breuer, K. *et al.* InnateDB: systems biology of innate immunity and beyond--recent updates and continuing curation. *Nucleic Acids Res* **41**, D1228-33 (2013).

77. Szklarczyk, D. *et al.* STRING v10: protein-protein interaction networks, integrated over the tree of life. *Nucleic Acids Res* **43**, D447-52 (2015).
